# Label- and Slide-Free Multispectral Quantitative Epi-Illumination Deep-Ultraviolet Microscopy

**DOI:** 10.34133/bmef.0337

**Published:** 2026-10-30

**Authors:** Mingxuan Si, Viswanath Gorti, Aaron D. Silva Trenkle, Arjun Renjith, Brienna E. Heinsz, Kyan Nguyen, Gabriel A. Kwong, Francisco E. Robles

**Affiliations:** ^1^ Wallace H. Coulter Department of Biomedical Engineering, Georgia Institute of Technology and Emory University, Atlanta, GA, USA.; ^2^ Department of Imaging Sciences, St. Jude Children’s Research Hospital, Memphis, TN, USA.

## Abstract

**Objective and Impact Statement:** We present a low-cost, LED-based, epi-illumination deep-UV microscope (epi-DUV) for rapid, label- and slide-free quantitative imaging of fresh bulk tissues, offering a powerful tool for point-of-care and intraoperative histological assessment. **Introduction:** Label- and slide-free imaging is highly desired in clinical pathology to avoid time-consuming tissue processing while preserving molecular information. Deep-UV microscopy offers high-resolution molecular contrast via intrinsic biomolecular absorption, but prior implementations were limited to thin sections and cell monolayers. **Methods:** We use an epi-DUV system to capture 255- and 280-nm absorption images, alongside tryptophan autofluorescence, from fresh, unprocessed bulk tissue. A deterministic transformation is applied to the 255-nm absorption images to generate virtual histological staining computationally. **Results:** This method yields quantitative maps of nucleic acid mass, protein mass, and quantum yield, while the transformation produces accurate H&E-like contrast. The system achieves 0.5-μm lateral resolution and ~6-μm effective slice thickness across a 707 × 707-μm field of view with ~330-ms exposure. This is the first demonstration of quantitative deep-UV molecular imaging of fresh, unlabeled bulk tissue. **Conclusion:** These results establish a simple, low-cost platform for rapid histological assessment of unprocessed tissue. By providing complementary molecular readouts alongside morphological contrast, this approach overcomes conventional processing limitations for rapid clinical applications.

## Introduction

Formalin-fixed and paraffin-embedded (FFPE) tissue processing remains the gold standard for histopathological examination, enabling diagnosis of a variety of diseases, including cancers, infections, and many other abnormalities [1–3]. FFPE relies on fixing tissues, embedding in paraffin wax, preparing thin tissue sections mounted on glass slides, followed by staining [e.g., hematoxylin and eosin (H&E)] to reveal tissue morphology (and in some cases, molecular composition) with high contrast. While these techniques provide high diagnostic utility and have been a mainstay tool in clinical pathology, they are time- and labor-intensive, requiring specialized equipment, trained personnel, and several chemical processing steps. The workflow, from sample acquisition to pathological evaluation, can take many hours to days [3–5], limiting intraoperative or point-of-care decision-making, which ultimately delays patient care. Although frozen sections provide a faster alternative to FFPE sections, with turnaround times of 20 to 30 min, their diagnostic quality is substantially lower [3,6,7]. Furthermore, these analyses cannot be performed in low-resource settings without access to histology laboratories and the required equipment and supplies.

Slide-free histology has recently emerged as a promising alternative, offering the potential for direct point-of-care imaging of intact bulk tissues with no or minimal processing. This can enable real-time histological evaluation, reducing time to diagnosis while preserving tissue for downstream analyses. Several optical techniques have been applied for slide-free histology, including point scanning methods such as confocal microscopy [8–11] and nonlinear multiphoton microscopy [12–15], which require some tissue processing for fluorescence labeling. More advanced label-free methods have also been introduced, such as stimulated Raman scattering [16–20] and photoacoustic microscopy (PAM) [21–28]. These techniques provide 3-dimensional (3D) histological images of un- (or minimally) processed tissues, but their clinical translation/adoption is hampered by the complexity, expense, and relatively large footprint of the systems. Wide-field imaging methods, including microscopy with ultraviolet surface excitation (MUSE) [29], fiber optic microscopy with ultraviolet surface excitation (FUSE) [30], and structured illumination microscopy (SIM) [31,32], have also been applied for slide-free imaging. These methods use much simpler instrumentation (i.e., lower costs, complexity, and footprint) with spatial multiplexing capabilities that offer faster acquisition times. However, these methods also require fluorescence labeling, which necessitates some degree of sample preparation. While fluorescence labeling is not as onerous a process as the entire FFPE and staining pipeline, it does introduce other caveats, including variable uptake of the fluorescent agent (particularly problematic for fresh tissues) and signal degradation via photobleaching. (Typically, tissues have to be fixed for optimal dye uptake, which adds to the processing time.) Furthermore, the very need for the fluorescent compounds and other necessary reagents can be a substantial added expense when imaging large volumes of tissues. Therefore, it is clinically desirable to have an imaging modality that is not only slide-free, fast, compact, and low cost but also label-free.

Deep-ultraviolet (UV) microscopy has recently emerged as a powerful approach for wide-field label-free molecular imaging [33–43]. By leveraging the intrinsic absorption properties of biomolecules, such as nucleic acids and proteins, in the deep-UV region of the spectrum (200 to 300 nm), this technique enables high-resolution, label-free, wide-field molecular imaging with simple and low-cost instrumentation. Deep-UV microscopy has been recently demonstrated for hematology [44] and histopathology applications, including label-free molecular analysis of prostate tissue sections [35,36]. One of the key advantages of deep-UV microscopy is its ability to enable quantitative mapping of biomolecules, providing pixel-level estimates of nucleic acid and protein mass with femtogram sensitivity derived directly from the absorption properties of these biomolecules [41,45]. By transforming qualitative images into quantitative maps, quantitative deep-UV microscopy has the potential to enable objective molecular phenotyping and real-time point-of-care assessment of the cellular and molecular composition, serving as an auxiliary readout for cytology and histology applications. However, prior implementations of deep-UV microscopy operate in transmission, hindering their applicability to unprocessed thick tissues.

Here, we introduce multispectral epi-illumination deep-UV microscopy (epi-DUV), a simple and low-cost method for rapid, label-free molecular imaging of fresh, unprocessed, bulk tissues. By way of multiple scattering within the thick tissue, in combination with the strong UV absorption, the acquired epi-DUV images effectively resemble transmission-based images of the tissue surface. Similar to transmission-DUV, the absorption images can be used to quantify nucleic acid and protein mass with femtogram sensitivity, as well as quantum yield. Moreover, absorption images acquired with 255-nm illumination can be deterministically mapped to an RGB color space to yield H&E-like contrast without the use of artificial intelligence (AI). We demonstrate the utility of the approach by imaging fresh murine and human tissue samples. To the best of our knowledge, this is the first demonstration of quantitative deep-UV molecular imaging of fresh, bulk tissues. Together, our system provides quantitative molecular information and H&E-like contrasts of unlabeled and unprocessed bulk tissues using a simple and low-cost wide-field system, offering a powerful and accessible tool for quantitative, stain- and slide-free histology.

## Results

### Histological imaging with epi-DUV imaging system

The epi-DUV system (Fig. 1A) is equipped with 4 narrow-band light-emitting diodes (LEDs), 2 with a center wavelength of 255 nm and 2 at 280 nm. The LEDs are directed to illuminate the bottom of the sample (which is placed atop a quartz slide) through a fused-silica light rod that acts as a light guide (Fig. 1B and C). The LEDs with matching wavelengths and their respective light guides are oriented opposite to each other as shown in Fig. 1C (blue arrows for 255-nm LEDs’ light guides; purple arrows for 280-nm LEDs’ light guides). This orientation increases the power (6.3 mW at the sample) and illumination homogeneity at the sample.

**Fig. 1.**
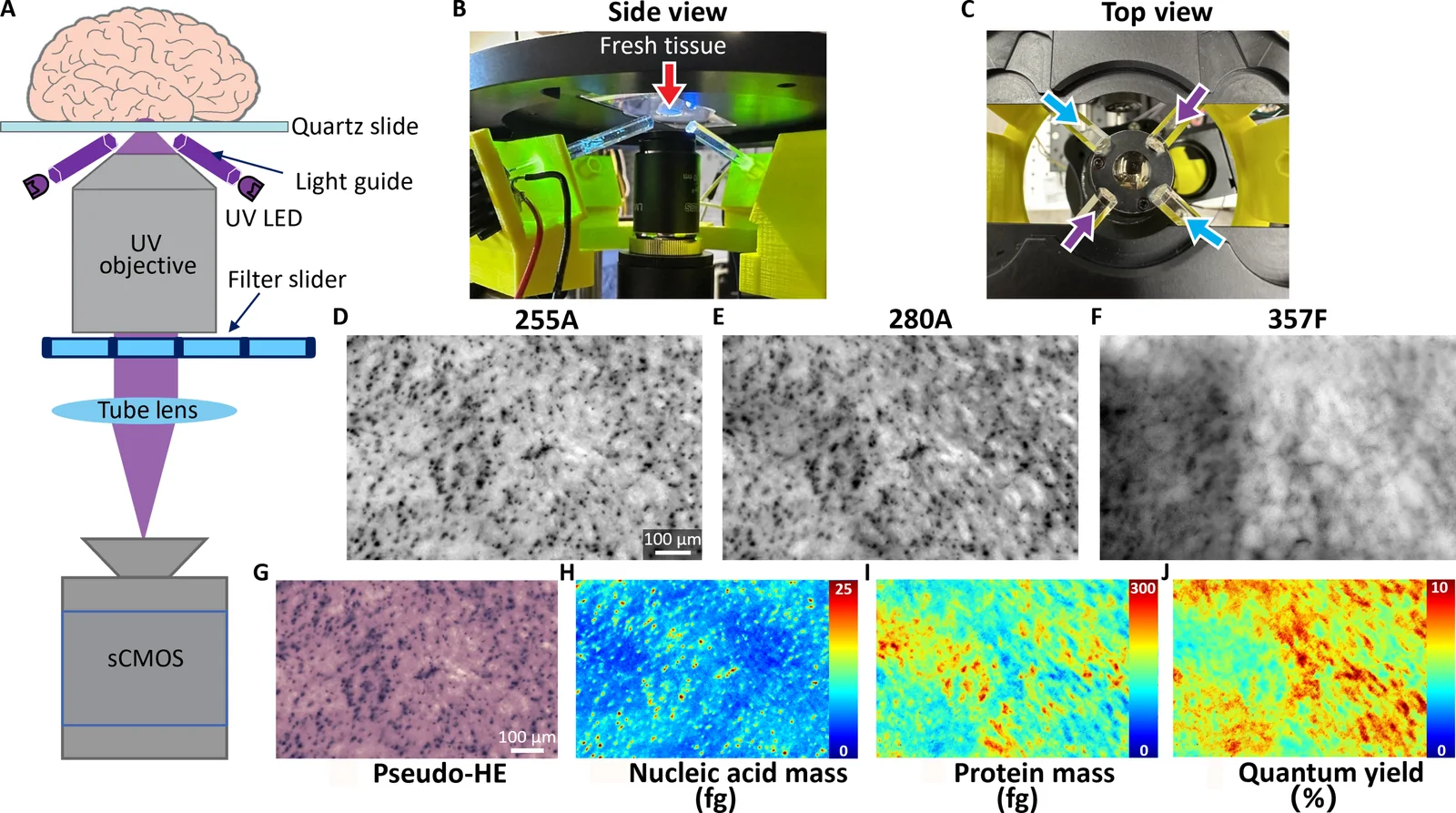
Epi-DUV imaging system setup and representative images from fresh mouse brain sample 1. (A) Schematic of the epi-DUV system, showing only 2 LEDs for better visualization. (B) Side view from just underneath the sample stage, showing only 2 LEDs for better visualization. Red arrow indicates the location of the fresh tissue. (C) Top view from above the sample stage. Blue arrows correspond to the 255-nm LEDs’ light guide. Purple arrows correspond to the 280-nm LEDs’ light guide. (D) Absorption image with 255-nm illumination and detection. (E) Absorption image with 280-nm illumination and detection. (F) Tryptophan autofluorescence image with 255-nm excitation and 357-nm detection. (G) Pseudo-H&E image of the same area using the 255A image only. (H) Nucleic acid mass map of the same area (in femtograms). (I) Protein mass map of the same area (in femtograms). (J) Tryptophan quantum yield map of the same area.

We generate image contrast based on either the tissue absorption or autofluorescence. We collect the 255-nm absorption (255A) and 280-nm absorption (280A) information by illuminating the sample with the two 255-nm LEDs and two 280-nm LEDs, respectively, and capturing the diffusely back-reflected light [46]. To collect the 2 absorption images, we also use bandpass filters (10-nm spectral bandwidth) centered at 255 and 280 nm, respectively, to remove any autofluorescence. Note that 255 and 280 nm correspond to the absorption peaks of nucleic acids and proteins [45,47]; thus, the absorption images (Fig. 1D and E) provide direct insight into their spatial distribution. We also collect autofluorescence information using the same 255-nm illumination for excitation, which provides additional contrast for fluorescent molecules. Since UV autofluorescence is dominated by tryptophan [48,49], we incorporate a bandpass filter centered at 357 nm, which corresponds to the tryptophan emission peak—this configuration selectively captures tryptophan autofluorescence. Note that 255 nm is not at the excitation peak of tryptophan [50], but this wavelength is chosen for excitation to generate negative contrast of cell nuclei due to their strong absorption at this wavelength (Fig. 1F).

From the 255A image alone, we generate pseudo-H&E images using RGB histogram matching between the measured absorption image and a reference H&E image (see Materials and Methods). This deterministic, pixelwise transformation preserves the spatial structure of the acquired 255A data and does not require training or use of AI. The generated pseudo-H&E images (e.g., Fig. 1G) exhibit comparable nuclear contrast and density to real histological images, both of which are essential for histological analysis.

To quantify molecular information (Fig. 1H to J), it is crucial to understand light-tissue interactions in the deep UV range. Tissue exhibits strong absorption due to the characteristic absorption peaks of nucleic acids and proteins [41,45]. Additionally, the scattering behavior of tissue in this range is assumed to follow Lambertian (isotropic) scattering at a depth near the surface. This behavior arises from a small anisotropy factor (*g* ~ <0.5) and a large scattering coefficient in biological tissues [51–54]. Under these conditions, it is reasonable to assume that a maximum of 2 scattering events occur before the scattering becomes fully isotropic.

We define the layer beneath the tissue surface where isotropic scattering occurs as the “diffusive layer”. Within the space between this layer and the tissue surface, isotropically scattered photons propagate back through the tissue surface again while undergoing substantial absorption. Photons that are not absorbed escape the tissue and are captured by the objective lens. We assume that the diffusive layer is so close to the tissue surface that light does not undergo an additional scattering event before exiting. In other words, we posit that the absorption coefficient in the deep UV range is comparable to, if not greater than, the scattering coefficient. This assumes that photons either escape the surface or are absorbed before contributing to the signal. These scattered photons can also be described as “reflected” from the diffusive layer, often referred to as “diffuse reflectance” in other studies [46,55,56]. This behavior allows us to apply Beer’s law to approximate tissue interactions and interpret our measurements as backscattering or diffuse reflectance originating from photons that were not absorbed within the diffusive layer. The diffusive layer can thus be treated as a homogeneous virtual light source within the tissue, denoted as $I_{0}$. It is important to note that while UV light can penetrate deeper into the tissue, photons scattering into these deeper regions are more likely to be absorbed than return to the surface and contributing to the detected signal. This provides tissue sectioning capabilities of the tissue surface layer.

Determining the exact distance between the diffusive layer and the tissue surface is challenging due to the limited availability of data on tissue properties in the deep UV range and the marked variability in reported values across studies [54,57,58]. In this work, we employ oblique illumination at ~70° to the optical axis to enhance optical sectioning in the UV range [59] and avoid detection of specular reflection from the quartz slide and tissue surface. Under this configuration, we assume that the distance between the diffusive layer and the tissue surface is comparable to the depth of focus (DOF) of our objective (~6 μm). This assumption is supported by the sharpness of our 255- and 280-nm images, which would appear blurry if the diffusive layer was substantially deeper than the DOF. Figure S2 further demonstrates this relationship, showing that illumination closer to the optical axis (45^o^ and 30^o^ instead of 70^o^) results in diminished image sharpness, reinforcing the connection between the depth of the diffusive layer and the DOF. A more comprehensive discussion on modeling tissue behavior in the deep UV region is provided in Materials and Methods and Text S1.

For molecular quantification, we adopt the 2-biomolecular-absorbing model of Zeskind et al. [45], where nucleic acids and proteins are the dominant absorbers at 255 and 280 nm. This model is reasonable even in complex tissues given that absorbance contributions from other biomolecules are minimal due to their low molar extinction coefficients or vastly lower concentrations (see additional discussion in Text S2). The virtual-source intensity, $I_{0}$, is estimated using known average nuclear concentrations of nucleic acids and proteins, and independently assessed with polystyrene beads (see Materials and Methods and Fig. S3). The calibrated $I_{0}$ is then used with Beer’s law to estimate nucleic acid and protein mass at each pixel. The tryptophan quantum yield map (Fig. 1J) is derived from the estimated protein concentration in conjunction with the tryptophan autofluorescence image (see Materials and Methods).

### Label-free epi-DUV absorption and autofluorescence imaging of fresh mouse brain

Figure 2 presents images of a fresh mouse brain acquired with the 3 independent channels: 255A (Fig. 2A), 280A (Fig. 2B), and tryptophan autofluorescence at 357 nm (357F) (Fig. 2C). The top row shows whole-brain views with colored boxes indicating 3 regions of interest. Subsequent rows correspond to zoomed-in views of the stratum pyramidale of the hippocampus (Fig. 2D), lateral thalamus (Fig. 2E), and pretectal thalamus (Fig. 2F), based on corresponding regions in the whole mouse brain atlas [60]. Across all regions, the 255A channel provides the strongest nuclear contrast and reveals very fine structural detail, consistent with the absorption peak of nucleic acids near 255 nm. For example, densely packed cells surrounding the hippocampus with clear nuclear contrast (red arrow) and the structure of a more myelinated region (black arrow) are clearly visible. The 280A channel shows slightly reduced nuclear contrast but emphasizes protein absorption. This difference is particularly evident in the pretectal thalamus (Fig. 2F), where some of the darker, more absorptive areas in 280A deviate from those observed in 255A. This can be attributed to variations in protein distribution across different regions of the mouse brain, such as dopamine receptor proteins (D1), which are highly concentrated in the thalamus but not the hippocampus [61]. The 357F channel provides the least nuclear contrast but highlights regions with strong tryptophan autofluorescence, reflecting high local tryptophan concentrations, such as in the hypothalamus [62]. The reduced nuclei contrast in the autofluorescence images is likely caused by a mismatch between the UV sectioning thickness and the DOF of the objective. Specifically, while DUV light that penetrates beyond the DUV “diffusive” layer is nearly completely attenuated (and thus never captured by the objective, restricting the detected signal to a very shallow surface layer), autofluorescence generated at these deeper regions will have a longer wavelength and will not be as efficiently attenuated, thus generating more out-of-focus signal. Therefore, the epi-DUV images natively have better sectioning capabilities than autofluorescence (and also fluorescence if the tissue was labeled). To further validate this, we conducted MUSE imaging using a nuclear stain (Hoechst), the results of which are included in Fig. S6. We used 280-nm excitation and >425-nm detection using the same monochrome camera used for epi-DUV as well as the same exposure time (330 ms). The results show very similar structure, except that nuclei have positive contrast in MUSE instead of negative contrast in epi-DUV, and importantly, we observed increased cellularity in the MUSE images of mouse brain, meaning the nuclei are more crowded, which suggests a thicker effective axial section/resolution in MUSE. This demonstrates that epi-DUV imaging achieves a shorter optical section than MUSE at the same illumination obliquity. This aligns with findings by Ching-Roa et al. [63], who observed a similar phenomenon when comparing MUSE with glycerol immersion (shorter optical section) versus water and air immersion (longer optical section), noting that the latter provided higher cellularity. We also observed more and sharper structural features in the mouse kidney in the 255A epi-DUV image than in the MUSE image. These results support our conclusion that epi-DUV provides a shorter effective sectioning thickness, and thus better axial resolution, than wide-field fluorescence signals (MUSE and also autofluorescence). A thinner sectioning thickness offers several distinct advantages. It improves axial resolution and yields an appearance closer to conventional 5-μm histological sections. Furthermore, it prevents the blurring that occurs when the optical sectioning thickness exceeds the objective’s DOF [63].

**Fig. 2.**
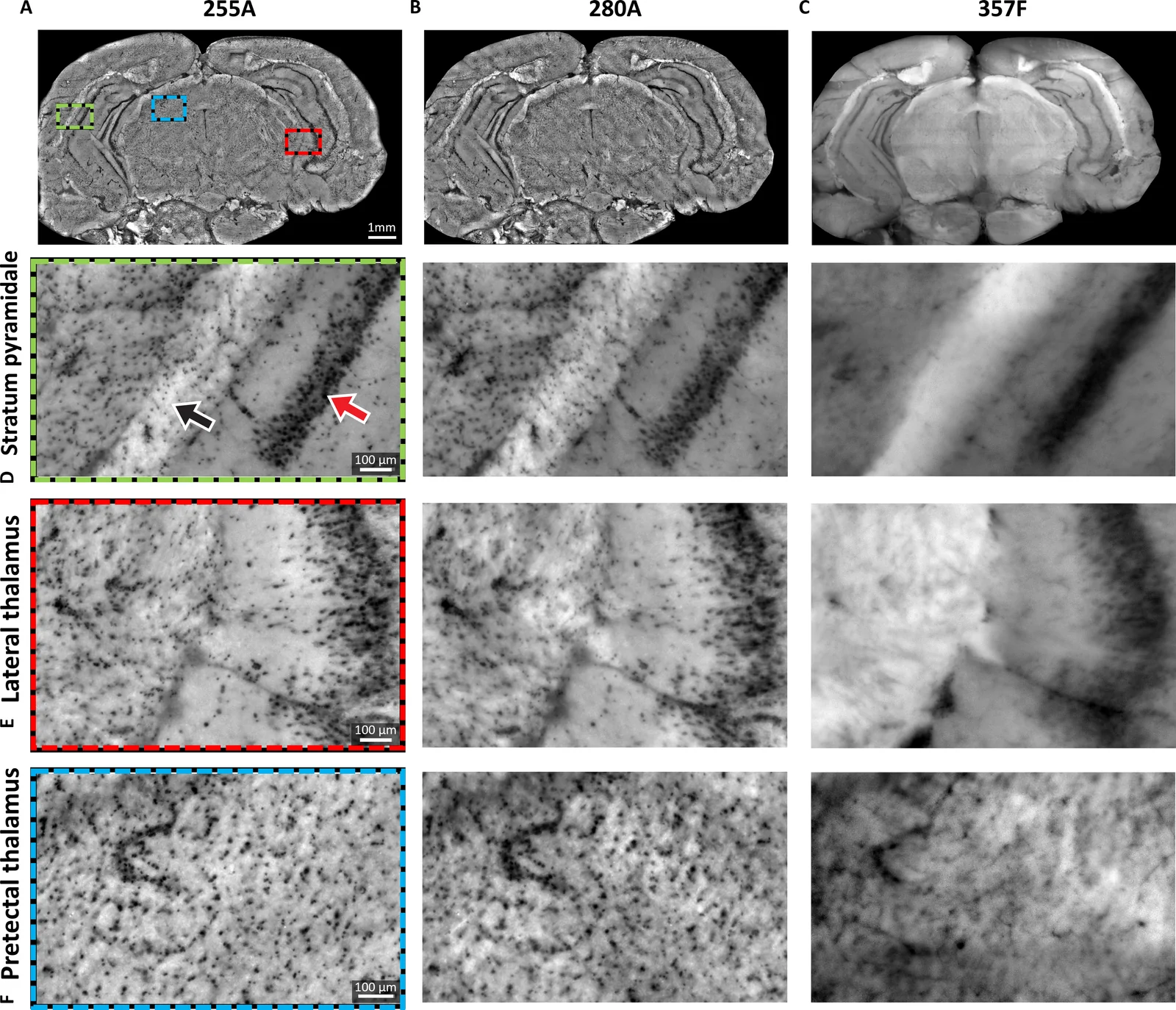
Label-free epi-DUV absorption and tryptophan autofluorescence images from fresh mouse brain. (A) Column for 255A images. (B) Column for 280A images. (C) Column for 357F images. (D) Row for zoomed-in region representing the stratum pyramidale of the hippocampus. The red arrow corresponds to densely packed cells surrounding the hippocampus. The black arrow corresponds to a more myelinated region. (E) Row for zoomed-in region representing the lateral thalamus. (F) Row for zoomed-in region representing the pretectal thalamus.

### Quantitative maps of representative regions in the fresh mouse brain

The quantitative maps are generated from the label-free epi-DUV absorption images and the autofluorescence images using Beer’s law. Figure 3 presents quantitative maps for the zoomed-in regions from Fig. 2 in the same order. Specifically, Fig. 3A, 3B, and 3C respectively show the nucleic acid mass, protein mass, and quantum yield of tryptophan for the stratum pyramidale of hippocampus (Fig. 3D), lateral thalamus (Fig. 3E), and pretectal thalamus (Fig. 3F). The nucleic acid and protein mass maps reveal nuclei and protein-rich domains, respectively. Notably, the protein mass map also highlights certain nuclei, reflecting the protein content within nuclei, but with lower clarity than the nucleic acid map, as the protein map emphasizes overall protein distribution across the tissue. Quantum yield map highlights areas with high tryptophan autofluorescence efficiency. The resulting quantitative values are consistent with prior reports from UV transmission imaging on live cells [41,45]. Specifically, the nucleic acid mass and protein mass, measured here, are up to 30 and 450 fg, respectively, per projected pixel in the object space (0.062 μm^2^) and a quantum yield of 15%. When scaled to the pixel size used in this study (see Materials and Methods), these values are in excellent agreement with those reported in the literature for live cells [41,45], which show a range of max mass values of 17 to 50 fg for nucleic acid, 170 to 620 fg for protein, and up to 20% quantum yield. The agreement in the average and range of masses helps support the validity of the simple epi-DUV 2-biomolecular-absorbing model for thick tissues.

**Fig. 3.**
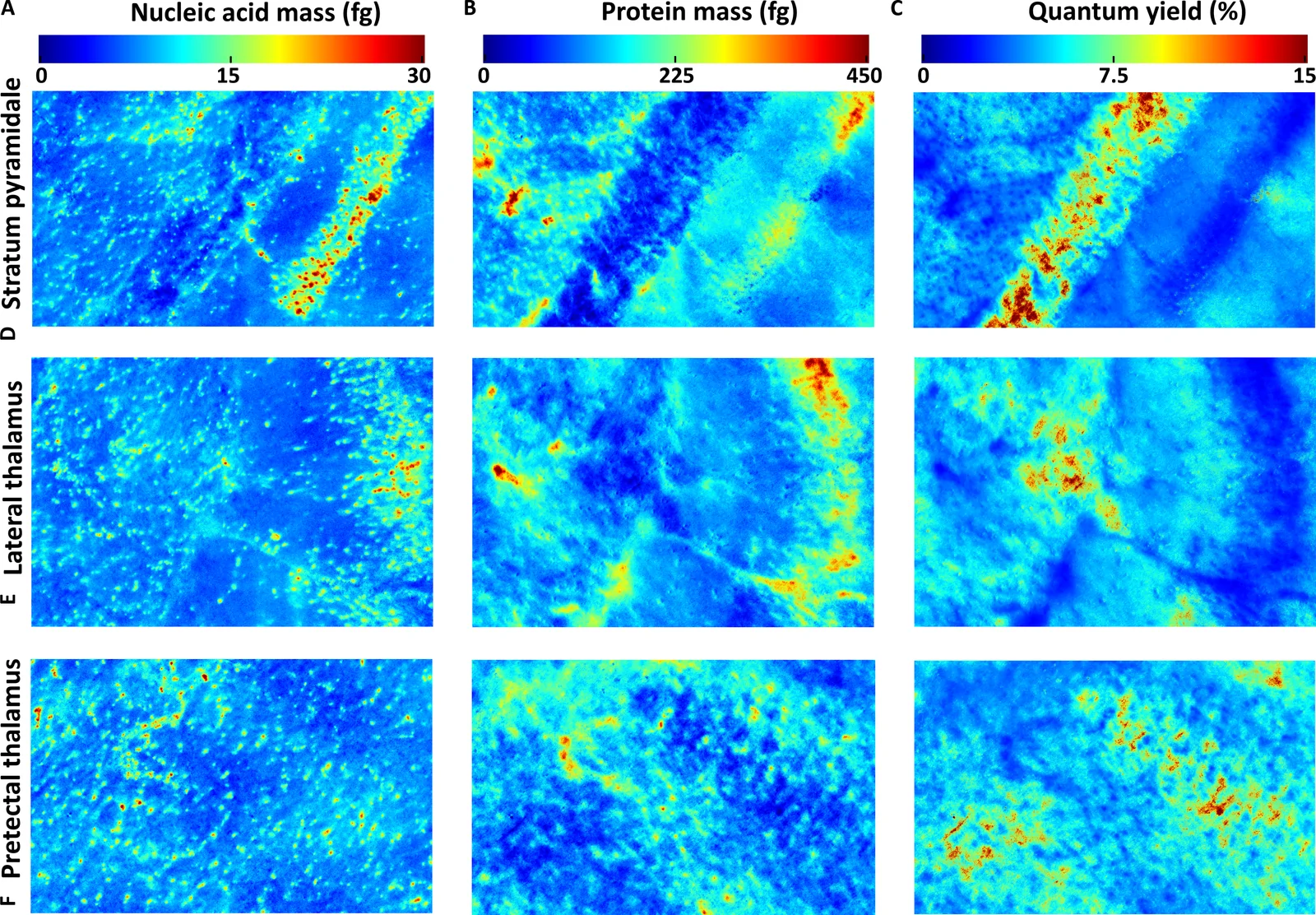
Quantitative maps of zoomed-in regions in Fig. 2 from fresh mouse brain sample 1. (A) Column for nucleic acid mass in femtograms. (B) Column for protein mass in femtograms. (C) Column for quantum yield of tryptophan in percentage. (D) Row for a region representing the stratum pyramidale of the hippocampus. (E) Row for a region representing the lateral thalamus. (F) Row for a region representing the pretectal thalamus.

### Pseudo-colorization via single-channel histogram matching

Beyond providing quantitative values from label-free epi-DUV images, we also apply a single-channel, fast transformation from the 255A image (Fig. 4A) to a pseudo-H&E image (Fig. 4B). The method uses an efficient and simple histogram-matching transformation to align the 255A image to each RGB channel of a reference H&E image (Fig. 4C) and then concatenates the 3 matched color-channel images (all derived from 255A images only). Prior work using dark-field reflectance ultraviolet microscopy (DRUM) [46] also produced pseudo-colorized images by combining 255A and 357F data with optical-density coefficients of H&E derived from Ruifrok and Johnston [64] (Fig. 4D). Compared with DRUM, our method offers (a) greater speed and simplicity by requiring only the 255A channel (no autofluorescence), (b) higher nuclear contrast and improved color rendition, and (c) flexibility to match any desired H&E color palette without using fixed transformation coefficients. Figure 4A to D shows representative figures of each image mode, with Fig. 4E to H illustrating one zoomed-in hippocampus region. Note the similar color tone between our pseudo-H&E image derived from the 255A image alone (Fig. 4B and F) and the real H&E image (Fig. 4C and G), both with clear nuclear contrast. Pseudo-H&E images derived from 255A and 357F (Fig. 4D and H) exhibit a large color tone mismatch and a blurrier background, due to the use of the fluorescence image as a counterstain. Additional images showing a myelinated region (Fig. 4I to L) and posterior thalamus region (Fig. 4M to P) further support the superior color transformation from our single-channel 255A images. We also note that because every output pixel in the pseudo-H&E image is derived directly from the corresponding measured 255A image, the transformation does not synthesize new spatial features.

**Fig. 4.**
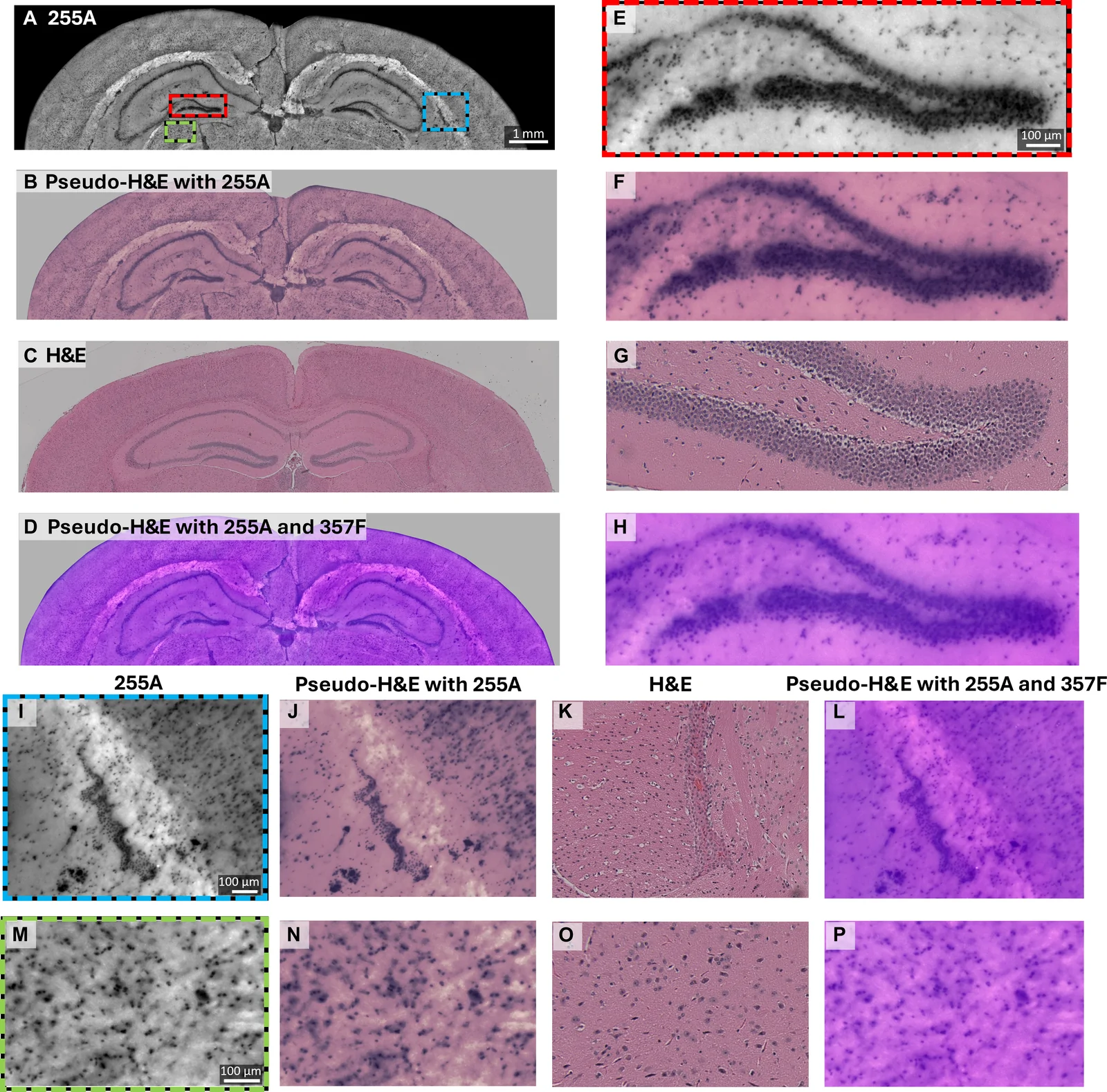
Pseudo-H&E colorization using single-channel 255A transformation and comparison to DRUM pseudo-H&E colorization. (A) 255A image of the mouse brain. (B) Pseudo-H&E image generated with 255A. (C) Brightfield image of an adjacent H&E-stained thin tissue section. (D) Pseudo-H&E image generated with both 255A and 357F (DRUM method). (E) Zoomed-in regions of 255A showing nuclei surrounding the hippocampus. (F to H) Pseudo-H&E transformed with 255A, adjacent H&E-stained thin tissue section, and DRUM pseudo-H&E of the hippocampus region, respectively. (I) 255A of another zoomed-in region from a myelinated area. (J to L) Pseudo-H&E transformed with 255A, adjacent H&E-stained thin tissue section, and DRUM pseudo-H&E of the myelinated area, respectively. (M) 255A of another zoomed-in region corresponding to the posterior thalamus. (N to P) Pseudo-H&E transformed with 255A, adjacent H&E-stained thin tissue section, and DRUM pseudo-H&E of the posterior thalamus region, respectively.

### Label-free epi-DUV images, quantitative maps, and pseudo-H&E of mouse kidney and human brain tumor samples

Beyond imaging the mouse brain, we apply our approach to mouse kidney and human brain tumor samples. The 255A (Fig. 5A) and 280A images of a cross-section of the surface of a whole mouse kidney demonstrate high nuclear contrast within the kidney tissue, with the pseudo-H&E image (Fig. 5B) closely resembling the adjacent H&E-stained slice (Fig. 5C). Notably, multiple glomeruli (black arrows) are distinctly visible in both the 255A and pseudo-H&E images (Fig. 5D), which are further confirmed by the adjacent H&E-stained thin slice of the same region (Fig. 5E), also showing clear glomerular structures (black arrows). Likewise, in the medulla region of the kidney, both the 255A and pseudo-H&E images (Fig. 5F and G) exhibit pronounced nuclear contrast, which is validated by an adjacent H&E-stained image of the same region (Fig. 5H). A zoomed-in view of a single glomerulus further highlights the nuclei contrast in the 255A (Fig. 5I) and pseudo-H&E images (Fig. 5J), which is again corroborated by the adjacent H&E-stained image of the glomerulus (Fig. 5K). Interestingly, the nucleic acid map (Fig. 5L) does not delineate the structure of the glomerulus as effectively as the protein mass map (Fig. 5M) and the quantum yield map (Fig. 5N). Notably, we find that the maximum protein mass is higher in kidney than in mouse brain and, similarly, that the average protein mass in kidney is higher than in the brain, which is consistent with prior reports [65]. This is further supported by comparing the average protein mass per unit area for mouse brain and mouse kidney samples, with results reported in Fig. S5.

**Fig. 5.**
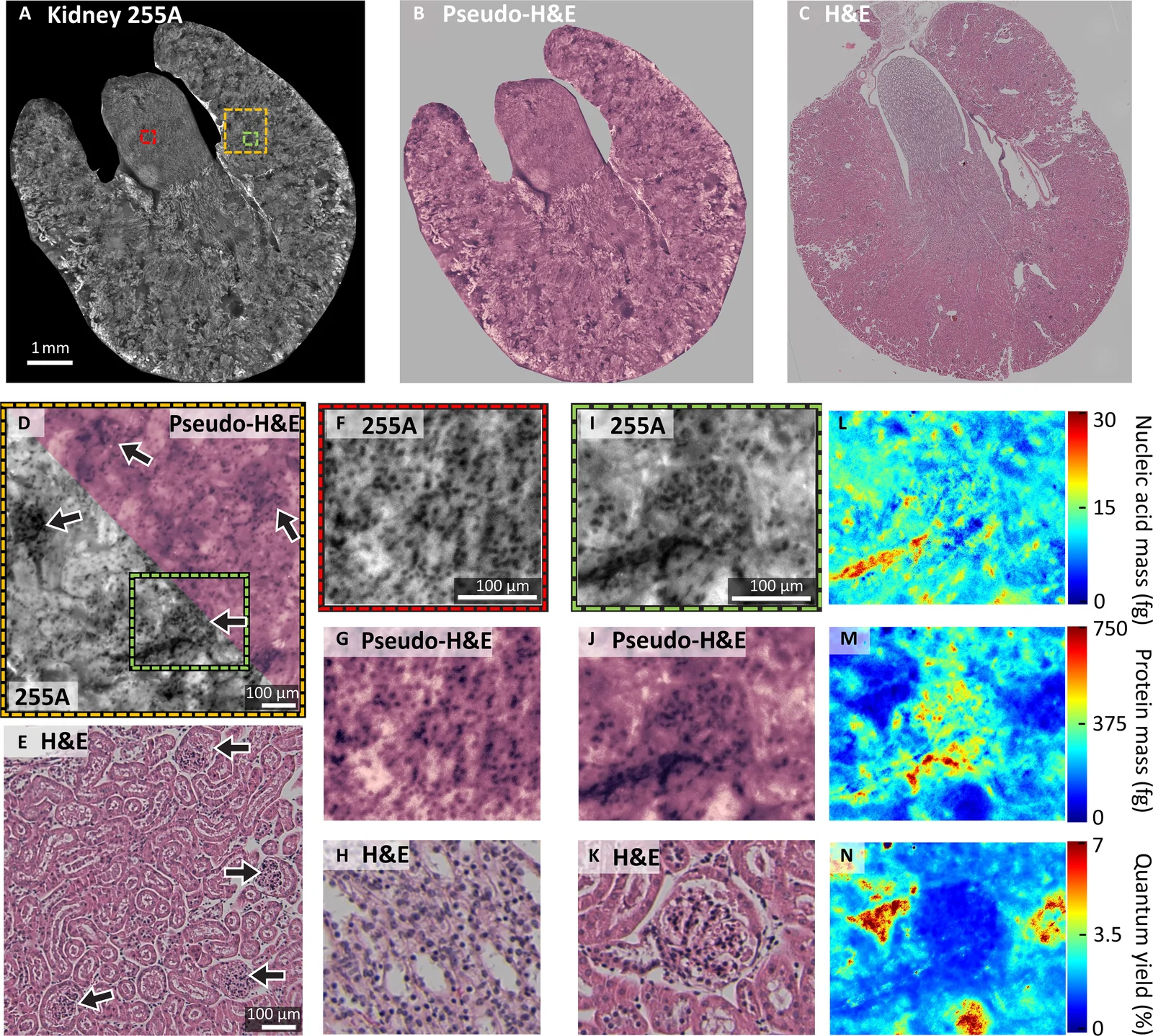
Epi-DUV images, pseudo-colorization, and quantitative maps of a mouse kidney sample. (A) 255A image of the whole mouse kidney sample. (B) Pseudo-H&E image of the kidney sample. (C) Adjacent H&E slice image of the kidney sample. (D) A zoomed-in region of (A), displaying multiple glomeruli (black arrows) in half 255A and half pseudo-H&E. (E) Adjacent H&E slice image with the same area, showing glomeruli (black arrows). (F) 255A image of another zoomed-in region of (A), representing the medulla of the kidney. (G and H) Pseudo-H&E and an adjacent H&E slice of this medulla region, respectively. (I) A zoomed-in region of (D), isolating a single glomerulus. (J and K) Pseudo-H&E image of the single glomerulus and an adjacent H&E slice of a single glomerulus. (L to N) Maps of nucleic acid mass, protein mass, and quantum yield of tryptophan of the single glomerulus image.

We also quantify the molecular mass maps of a human brain tumor sample (astrocytoma, grade 4). The images clearly exhibit densely packed cell nuclei, which aligns with diagnostic indicators of malignancy, such as uncontrolled cell proliferation and nuclear pleomorphism (Fig. 6A). A zoomed-in region (Fig. 6B) reveals clear nuclear contrast in the 255A image, the pseudo-H&E image, and nucleic acid mass map (Fig. 6C). A further zoomed-in region (Fig. 6D and E) highlights a dense tumor region, which is validated by the adjacent H&E-stained slice (Fig. 6F). Interestingly, the nucleic acid mass distribution (Fig. 6G) differs substantially from the protein mass distribution (Fig. 6H). This observation indicates that proteins in this malignant region are not predominantly localized within the cell nuclei, suggesting altered protein distributions in cancer [66]. Additionally, this region exhibits both lower maximum and average quantum yield values (Fig. 6I) compared to the mouse brain, a difference that will be further analyzed in Fig. 7.

**Fig. 6.**
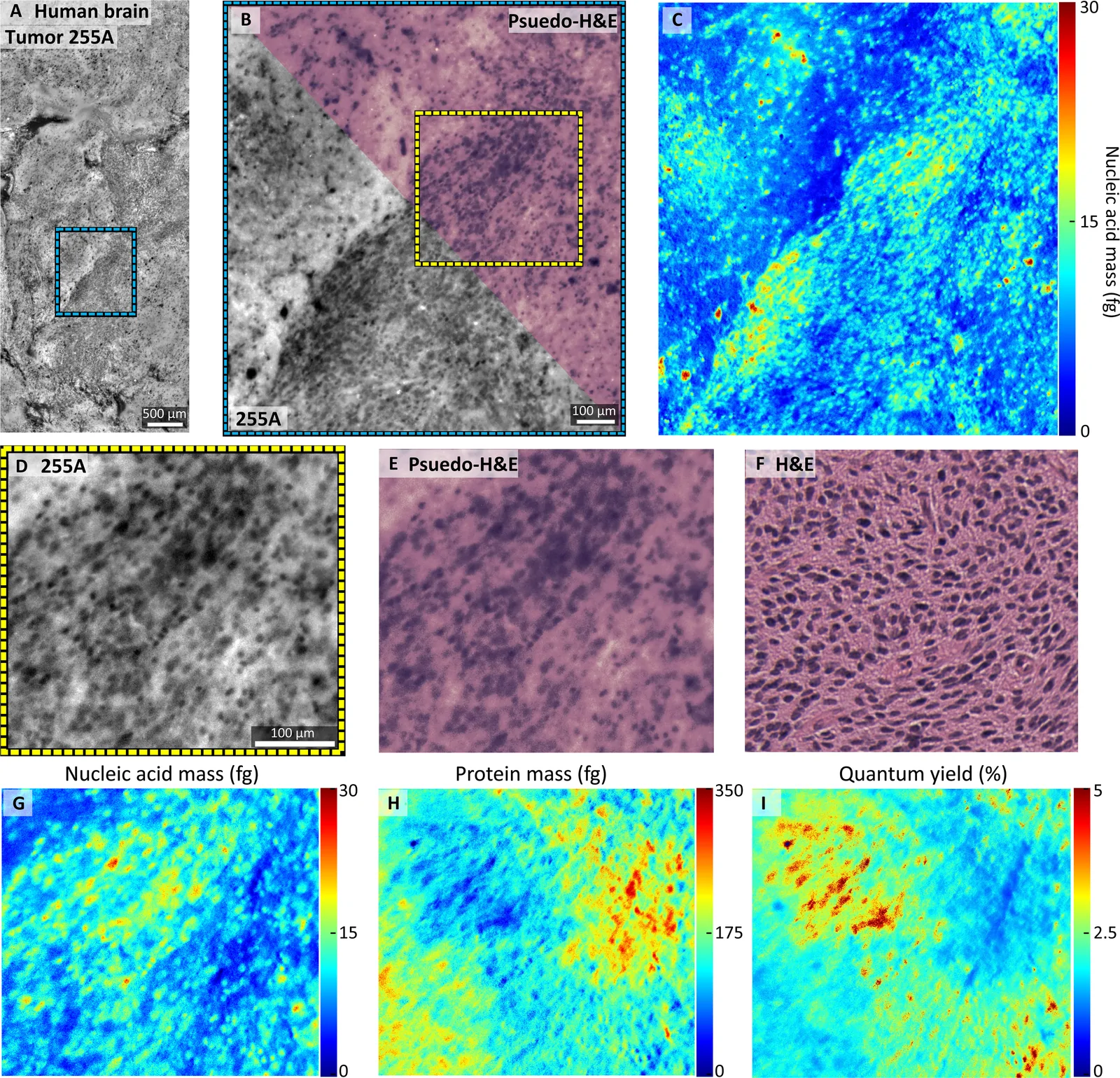
Epi-DUV images, pseudo-colorization, and quantitative maps of a human brain tumor sample. (A) 255A image of a brain tumor region. (B) A zoomed-in region corresponding to a dense tumor region. (C) The nucleic acid mass map of the region in (B), highlighting regions with high nuclear density and concentration of nucleic acid. (D) 255A image of a further zoomed-in region in (B). (E) Pseudo-H&E image of (D). (F) Adjacent H&E slice image of (D). (G to I) The nucleic acid mass map, protein mass map, and quantum yield of tryptophan of the region in (D).

**Fig. 7.**
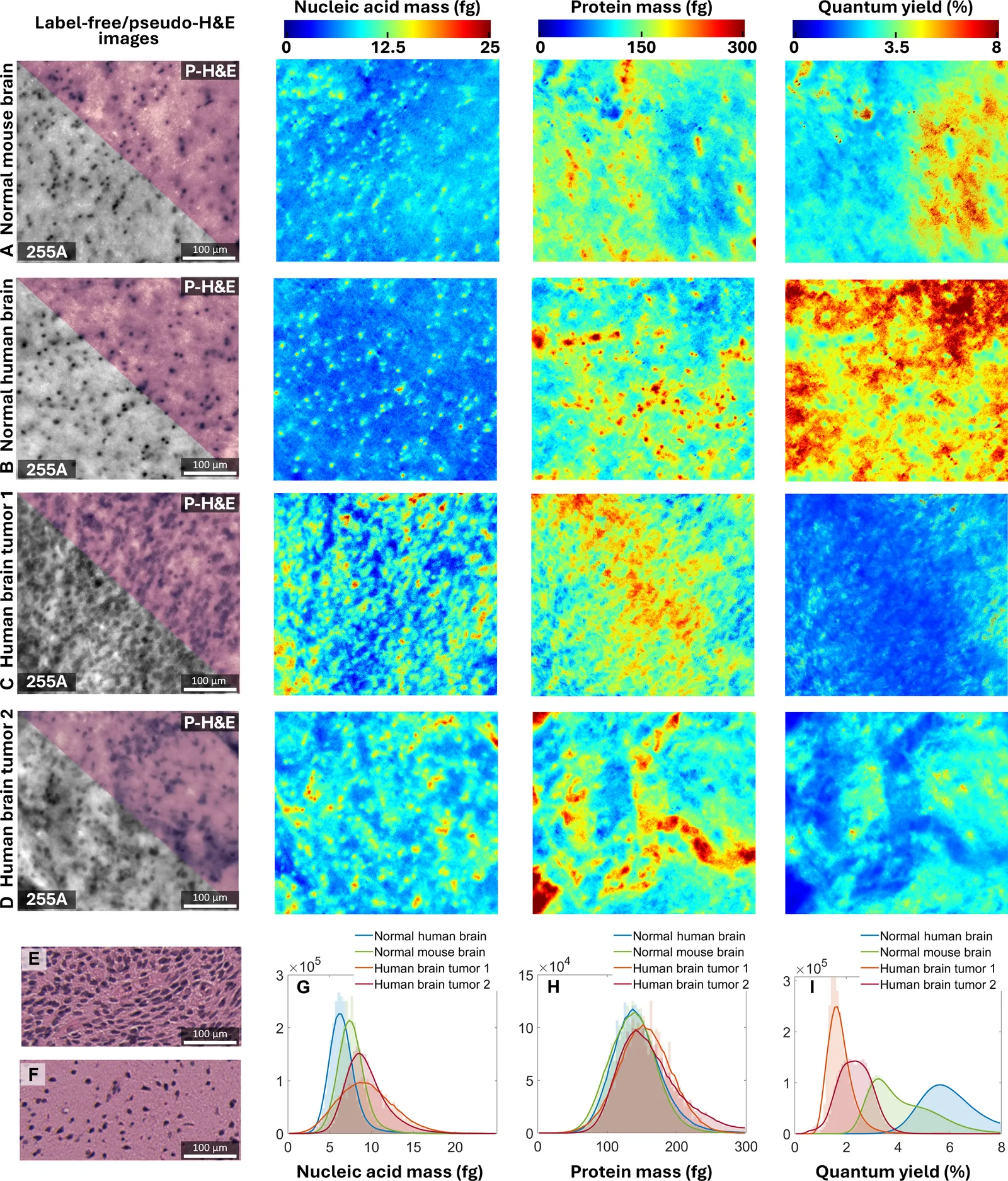
Comparison of quantitative values for brain tissues. (A) Row representing a cortex region in normal mouse brain, comprising (from left to right) 255A image with pseudo-H&E image (combined into one figure), nucleic acid mass, protein mass, and quantum yield. (B) Row representing a cortex region in a normal human brain in the same sequence as (A). (C and D) Row representing a cancerous region from 2 separate glioma samples in the same sequence as above. (E) Adjacent H&E slice image of the human brain tumor 1 sample. (F) Adjacent H&E slice image of the normal mouse brain cortex. (G to I) Histogram of nucleic acid mass, protein mass, and quantum yield, respectively, of the 4 regions.

### Comparison of quantitative values for brain tissues

This section demonstrates how quantitative data can be utilized to compare brain tissue disease status. We present a comparison of a healthy mouse brain cortex region (Fig. 7A), a healthy human brain cortex region (Fig. 7B; adjacent tissue from a tumor excision), and tumor regions from 2 glioma patients (Fig. 7C and D). The cellularity of these images is corroborated by adjacent H&E images of a tumor region (Fig. 7E) and healthy mouse brain cortex (Fig. 7F).

The quantitative values reveal intriguing molecular differences between these tissues. For instance, the nucleic acid and protein mass distributions for the normal mouse and human brain cortex regions show notable similarities (see histograms, Fig. 7G and H). In contrast, the glioma regions (Fig. 7C and D) exhibit appreciably higher cellularity than the healthy human brain cortex (Fig. 7B), as expected. However, the nuclei in the glioma tissues not only are more dense but also display increased overall nucleic acid mass and a wider distribution compared to the healthy tissues (see histogram, Fig. 7G). Interestingly, the protein content does not differ substantially between the healthy and cancerous tissues, whereas the quantum yield demonstrates a pronounced variation. The histogram of the quantum yield for the 2 cancerous regions and the healthy human brain (Fig. 7I) shows that the average value in the human brain tumor is lower by more than half of the healthy tissue. This reduction may be attributed to differences in brain regions or alterations in the fluorescence properties of tryptophan due to the local tumor environment. This finding is supported by a recent study demonstrating that fluorescence intensity of endogenous fluorophores in glioblastoma regions is statistically significantly lower than that of healthy regions [67], which is directly related to the quantum yield. Although (to our knowledge) there is no existing literature directly characterizing changes in tryptophan quantum yield in tumor settings, our illustration underscores how quantitative differences can be applied to gain new insight into the molecular properties or composition of intact tissues and disease status.

## Discussion

In this study, we demonstrate label- and slide-free epi-DUV imaging of fresh, bulk tissue specimens using a low-cost, simple, and compact LED-based wide-field platform. The system provides high-contrast absorption and autofluorescence images from a shallow surface layer and yields estimates of nucleic acid mass, protein mass, and tryptophan quantum yield. To the best of our knowledge, this approach represents the first demonstration of quantitative deep-UV molecular imaging in bulk tissue samples using a simple widefield configuration. We also show that a single 255A acquisition can be mapped deterministically to an H&E-like image, providing more familiar readouts for clinicians compared to the native absorption images or molecular maps. Together, epi-DUV could serve as an accessible alternative to traditional histological workflows, providing clinicians H&E-like images with enhanced molecular information at the subcellular level without tissue processing or labeling.

For quantification, our approach assumes that nucleic acids and proteins are the only 2 biomolecules absorbing at 255 and 280 nm. This assumption is reasonable since these biomolecules are the dominant absorbers at these wavelengths, as has been reported in previous studies [41,45]. Indeed, other biomolecules present in tissues, such as lipids and coenzymes, absorb at these 2 wavelengths; however, their overall absorbance is minimal due to either low molar extinction coefficients or vastly lower concentrations, resulting in a negligible effect on our absorbance models (more details are provided in Text S2) [68–72]. Adding additional wavelengths, corresponding to the peak absorbance of other biomolecules, could provide additional and more precise molecular information.

While the use of Beer’s law is common to quantify deep-UV microscopy images, previous implementations have been strictly limited to thin tissue sections or cell monolayers, where measuring the initial light intensity ($I_{0}$) is trivial. Quantifying the molecular information in fresh, thick tissue is substantially more challenging, but the strong absorption and scattering properties of tissue in the UV spectrum still enable use of this simple model. In thick tissue, $I_{0}$ is not the initial intensity of the light source, but rather the intensity of light that is redirected toward the collection optics at the “diffusive layer” immediately below the surface. By leveraging a priori knowledge of the average molecular absorption of nuclei and additional independent validation using polystyrene beads embedded in thick tissue (detailed in the Quantification of nucleic acid and protein masses section and Fig. S3), $I_{0}$ can be estimated, enabling computation of the molecular composition of fresh tissue at the pixel level.

The ability to obtain pseudo-H&E images of bulk, fresh tissues deterministically from a single 255A acquisition is important. Indeed, this color transformation does not produce images identical to FFPE-processed H&E sections, but they recapitulate critical histological detail with a strong resemblance to the clinical standard. Results could be further enhanced by leveraging neural networks, and given the strong similarity between the pseudo-H&E and real H&E contrast, the results may be robust against hallucinations, which is the main risk of using AI for image translation. However, the improvement provided by deep learning networks may be marginal, as nuclear structures (size, shape, density, pleomorphism, and spatial organization) are some of the most critical features for tissue evaluation [73,74] and are clearly visible in the native UV absorption and the pseudo-H&E images. The RGB histogram matching approach used here is simple, fast, and robust. We also note that our 255A pseudo-H&E images are capable of capturing structurally diverse information. For example, the kidney sample (Fig. 5) contains highly complex and distinct structures, including glomeruli, various tubules, and differing cortical and medullary regions, all of which are clearly resolved. Because nuclei have a strong absorption peak at 255 nm regardless of the tissue type, nuclei will reliably map to dark purple (hematoxylin). Conversely, protein-rich structures such as epithelium, blood vessels, and fibrosis (collagen) absorb less at this wavelength and will naturally map to pink (eosin). Adipose tissue (fat) lacks these dense absorbers and will appear clear, similar to standard H&E. Therefore, the physical principles underlying our contrast generation naturally and accurately accommodate complex, heterogeneous tissue microenvironments.

As we have explored in previous work [35,36], we highlight that single-color (255A) DUV imaging of fixed tissue sections does not yield a realistic pseudo-H&E image, unlike fresh, unaltered tissues, indicating substantial molecular compositional differences between fresh, unaltered tissues and fixed tissue sections. Such molecular changes have also been observed under phase imaging where the refractive index composition of FFPE-processed tissues is substantially different than those of fresh tissues [75]. More recently, using mid-infrared spectrochemical imaging (MIRSI) [76], it was found that FFPE tissues have substantially altered chemical/molecular and morphological compositional differences compared to frozen sections and—in particular—fresh tissues. A similar effect has been reported in autofluorescence imaging using computational high-throughput autofluorescence microscopy by pattern illumination (CHAMP) [77], where thin tissue slices exhibited considerably higher nuclear contrast than corresponding fresh tissues. Figure S7 illustrates differences between transmission-DUV of FFPE thin tissue sections and epi-DUV imaging of thick fresh tissue, showing some differences in structure (similar to previous literature), but also exhibiting similar quantitative attenuation values for nuclei. A more comprehensive comparison between epi-DUV and transmission-DUV can be found in Text S3. Together, these findings underscore the importance of methods that can provide label-free structural and molecular information of intact/unaltered (fresh, unstained) tissues. The results of this work show that epi-DUV microscopy is ideally suited to address these challenges.

We note that several other slide-free methods have also been demonstrated. Reflectance confocal microscopy (RCM) [78], CHAMP [77], UV-PAM [79], photoacoustic/photon absorption remote sensing (PARS) [26,27,80,81], quantitative oblique back-illumination microscopy (qOBM) [82–87], MUSE, FUSE, and light-sheet-based UV microscopy approaches (UV-LS) [88,89] can produce histology-like images of unprocessed tissue. However, each has unique trade-offs. For example, label-free methods like RCM, CHAMP, PARS, UV-PAM, and UV-LS rely on expensive lasers which substantially increase the cost and complexity of the systems, and they utilize neural networks for virtual staining, which as discussed above are prone to hallucination errors. qOBM is also label-free and uses simple instrumentation with wide-field, LED-based illumination, but it similarly relies on neural networks for virtual staining. We also note that RCM and qOBM use near-infrared light and can thus provide 3D information beyond the very surface layer of the tissue. The capabilities of epi-DUV are similar to those of MUSE and FUSE: Both use simple, low-cost instrumentation with wide-field UV illumination. But epi-DUV has the important advantages of being label-free and enabling readouts of endogenous biomolecules. Another important advantage is the ability of epi-DUV to achieve thinner optical sectioning, and consequently, better axial resolution, compared to wide-field autofluorescence and fluorescence (see comparison of epi-DUV and MUSE in Fig. S6, and MUSE imaging protocol in Text S4).

In summary, we have developed a novel, LED-based epi-DUV microscopy system that enables rapid, label-free, and slide-free quantitative imaging of fresh, thick tissue samples. By leveraging the intrinsic absorption and autofluorescence properties of biomolecules in the deep-UV spectrum, we achieve subcellular resolution and generate quantitative mass maps of nucleic acids, proteins, and tryptophan quantum yield, alongside realistic pseudo-H&E images. Given its accessible instrumentation, simple implementation free of any sample preparation or staining, nondestructive (i.e., tissue-preserving) nature, and rich endogenous molecular contrast, epi-DUV microscopy has the potential to become an important tool for rapid, point-of-care histological analysis.

## Materials and Methods

### Label-free epi-DUV imaging system

The epi-DUV system is built on a conventional wide-field inverted microscope setup (Fig. 1A) with a modified illumination path incorporating LEDs and light guides (Fig. 1B). Narrow-band surface-mount LEDs with center wavelengths of 255 nm (Photon Wave Inc.) and 280 nm (Nichia Inc.) were used. These LEDs were mounted on heat sinks and secured below the sample stage using customized 3D-printed LED holders (Fig. 1B). Light pipes (63-093, Edmund Inc.) with a 4-mm aperture were inserted into the LED holders to guide the light from the LEDs, ensuring uniform illumination at the sample. The light pipes are oriented at a 70° angle to the optical axis, effectively operating in a dark-field configuration, as specular reflections fall outside the numerical aperture (NA) of the objective. The power of the two 255-nm LEDs and two 280-nm LEDs at the sample plane was controlled to be approximately equal (~7.1 mW). The system lateral resolutions at 255 and 280 nm were measured to be ~550 nm and ~600 nm, respectively, as detailed in Fig. S1.

Light information from the sample was collected using a UV-transmissive objective (LMU-15X-UVB, NA = 0.31, Thorlabs Inc.), passed through band-pass filters centered at 255, 280, and 357 nm (FF01-254/8-25, BrightLine Inc.; FF01-280/10, BrightLine Inc.; 18-384, Edmund Inc.), which were installed on a filter slider (CFS1, Thorlabs Inc.) and a UV-transmissive tube lens (*f* = 150 mm) and finally captured by a UV-sensitive scientific complementary metal-oxide semiconductor (sCMOS) camera (ATX081S-UC, 2,840 × 2,840 pixels, 2.74-μm pixel size, Lucid Inc.).

### Sample collection, image acquisition, and image processing

This study examined 3 types of tissues: mouse brain, mouse kidney, and human brain tumor. Mouse brain and kidney samples were obtained in accordance with the Institutional Animal Care and Use Committee of the Georgia Institute of Technology. Human brain tumor samples were obtained from the Winship Cancer Institute of Emory University under approved, institutional review board-exempt protocols. All tissues were de-identified and imaged fresh, typically within 6 h of removal. The 2 human brain tumor samples were confirmed post-surgery as a grade 4 astrocytoma with isocitrate dehydrogenase (IDH) mutation, classified according to the World Health Organization Classification of Tumors of the Central Nervous System.

For image acquisition, fresh tissue samples were placed between quartz slides to flatten the bottom surface, minimizing surface irregularities. The quartz slide was secured on a sample stage (MPRC & MPSH2, Thorlabs Inc.), connected to a 3-axis translation stage (LX30, Thorlabs Inc.). Tissue scanning was automated using stepper motors, motor drive shafts, and motor drivers controlled via a custom MATLAB application interfaced with a microcontroller (Arduino Mega 2560 REV3). The tissue was scanned with approximately 20% overlap between adjacent fields of view (FOVs) to facilitate image stitching. We note that the irregular surface of bulk tissues can result in the tissue surface being out of focus. Although this effect was not observed in this work, future systems could implement extended depth of focus (EDOF) to further improve image robustness against surface variations as has been done in other slide-free methods [90].

Each FOV measured ~707 μm × 707 μm, and the scanning time for a 10 mm × 10 mm tissue sample at an exposure time of ~330 ms was ~3 min per wavelength (a throughput of ~0.55 mm^2^/s). This throughput is comparable to that of a commercial digital scanner and could be optimized further by reducing the exposure (leveraging higher power LEDs), reducing scanning overlap, and synchronizing the LEDs and filters with image acquisition to capture the 3 wavelengths per FOV sequentially. It is worth noting that, even with our current parameters, our imaging speed is faster than standard frozen-section histology. More importantly, our approach does not suffer from freezing artifacts in frozen sections, which can markedly compromise diagnostic quality. Instead, epi-DUV delivers realistic H&E-like images, which typically require days to process via standard methods, alongside rich molecular information from fresh tissue, all well within the intraoperative time window.

To minimize stitching artifacts caused by uneven illumination during image stitching, each individual FOV is normalized using a background image. This background image is created by applying a severe Gaussian filter (σ=1,000 pixels) to each FOV, thus retaining only very low spatial frequency information. Dividing each FOV by its Gaussian-filtered version produces a normalized FOV with a highly uniform illumination distribution. This process has been referred to as flat-field correction [91].

H&E images for all tissue types were acquired digitally using a commercial slide and plate scanner at 10× magnification (NA = 0.3) (BioTek Cytation 7, Agilent Technologies Inc.).

### Quantification of nucleic acid and protein masses

As briefly described in the Results section, the quantitative mass maps were computed based on Beer’s law, assuming that a diffusive layer at a specific depth within the tissue acts as a virtual light source for UV illumination. This assumption, combined with the strong absorption characteristics of biological tissues in the deep-UV range, enables the behavior of light within the tissue to be modeled as predominantly absorptive within the diffusive layer. Consequently, the measurements in the 255A and 280A images primarily reflect photons that were not absorbed at these wavelengths, following principles similar to conventional transmission UV microscopy. Under this model, the absorbance at each wavelength can be expressed using Beer’s law [45], assuming that nucleic acids and proteins are the only biomolecules contributing to absorption at 255 and 280 nm:

$$\begin{array}{c} A_{\lambda }\left(x,y\right)=\\ \varepsilon_{\lambda }^{\text{protein}}\times c^{\text{protein}}\left(x,y\right)\times l_{\lambda }+\varepsilon_{\lambda }^{\text{nucleic acid}}\times c^{\text{nucleic acid}}\left(x,y\right)\times l_{\lambda } \end{array}$$ (1)

where $A_{\lambda }\left(x,y\right)$ is the wavelength-dependent absorbance at each pixel, $\varepsilon_{\lambda }^{\text{protein}}$ is the wavelength-dependent molar extinction coefficient of protein, $c^{\text{protein}}\left(x,y\right)$ is the concentration of protein at each pixel, $l_{\lambda }$ is the wavelength-dependent path length, $\varepsilon_{\lambda }^{\text{nucleic acid}}$ is the wavelength-dependent molar extinction coefficient of nucleic acid, and $c^{\text{nucleic acid}}\left(x,y\right)$ is the concentration of nucleic acid at each pixel. The path length is estimated to be approximately equivalent to the DOF of the objective, calculated to be ~6 μm [92]. As shown in Text S1, the UV penetration depth of diffusely reflected light can reasonably be assumed to be comparable to the DOF. If this assumption were incorrect, there would be substantial out-of-focus information within the FOV, which is not observed in the sharp, high-contrast 255A and 280A images, where nuclei are clearly visible. (In fact, with less oblique illumination, for example, using 45^o^ or 30^o^ instead of 70^o^, appreciably more out-of-focus background light is indeed observed—see Fig. S2.) Additionally, the relationship between penetration depth and DOF with respect to the focus quality of the image has been discussed in a prior study [77].

Absorbance can be rewritten as:

$$A_{\lambda }\left(x,y\right)=-{\text{log}}_{10}\left(\frac{I_{\lambda }\left(x,y\right)}{I_{0,\lambda }}\right)$$ (2)

where $\frac{I_{\lambda }\left(x,y\right)}{I_{0,\lambda }}$ is the wavelength-dependent transmittance at each pixel. $I_{\lambda }\left(x,y\right)$ is the wavelength-dependent transmitted intensity at each pixel, representing either the 255A or 280A images, and $I_{0,\lambda }\;$ is the wavelength-dependent intensity at the diffusive layer at each pixel (i.e., the intensity of the virtual light source). To find the pixel-wise concentration of protein and nucleic acid, we must first calibrate $I_{0,\lambda }$ of the epi-DUV system. It can be computed by assuming known average concentrations of protein and nucleic acid in cell nuclei, which are estimated from the literature to be ~300 mg/ml and ~30 mg/ml [45], respectively. The concentrations can be converted into molarity (M) based on the average molar mass of protein (52,728 Da) and nucleic acid (330 Da) [45]. With this, Eq. (1) can be rearranged to estimate $I_{0,\lambda }$:

$$\begin{array}{c} {\text{log}}_{10}\left(I_{0,\lambda }\right)=\\ \varepsilon_{\lambda }^{\text{protein}}\times c_{\text{nuclei}}^{\text{protein}}\times l_{\lambda }+\varepsilon_{\lambda }^{\text{nucleic acid}}\times c_{\text{nuclei}}^{\text{nucleic acid}}\times l_{\lambda }+{\text{log}}_{10}\left(I_{\lambda ,\text{nuclei}}\right) \end{array}$$ (3)

$I_{255,\text{nuclei}}$ and $I_{280,\text{nuclei}}$ represent the average values of over 50,000 segmented visible nuclei in the stitched 255A and 280A images of a mouse brain sample, respectively. Using these values and the quantities described in Table 1, $I_{0}\;$ at 255 and 280 nm can be calculated. In this study, $I_{0}$ is assumed to be constant for all tissues analyzed, as the absorption properties of these biological tissues are comparable [54]. Based on this assumption, we applied the calculated $I_{0}$ to independent mouse brain samples, mouse kidney, and human brain tumor tissues. We also repeated the procedure by calculating the quantitative values for the mouse kidney using the $I_{0}$ derived from independent kidney tissue, which produced results very similar to those obtained using the $I_{0}$ calculated from the mouse brain, as shown in Fig. S4.

**Table 1.** Quantities for computing mass maps and autofluorescence quantum yield of tryptophan. All molar extinction coefficients, $N_{\mathrm{aa}}$, and $F_{\mathrm{Trp}}$ were referenced from [45].

Quantities	Values	Units
$\varepsilon_{255}^{\text{protein}}$	36,057	$M^{-1}{\mathrm{cm}}^{-1}$
$\varepsilon_{280}^{\text{protein}}$	54,129	$M^{-1}{\mathrm{cm}}^{-1}$
$\varepsilon_{255}^{\text{nucleic acid}}$	7,000	$M^{-1}{\mathrm{cm}}^{-1}$
$\varepsilon_{280}^{\text{nucleic acid}}$	3,500	$M^{-1}{\mathrm{cm}}^{-1}$
$\varepsilon_{255}^{\mathrm{Trp}}$	3,787	$M^{-1}{\mathrm{cm}}^{-1}$
$c_{\text{nuclei}}^{\text{protein}}$	0.0057	M
$c_{\text{nuclei}}^{\text{nucleic acid}}$	0.091	M
$l_{255}$	5.84	μm
$l_{280}$	6.41	μm
$T_{\mathrm{AF}}$	330	ms
$T_{\text{trans}}$	10	ms
${\mathrm{TE}}_{\mathrm{AF}}$	0.75	
${\mathrm{TE}}_{\text{trans}}$	0.48	
NA	0.31	
$N_{\mathrm{aa}}$	466	
$F_{\mathrm{Trp}}$	0.014	

To further validate the calibrated $I_{0}$ from the mouse brain, we used 10-μm polystyrene beads. First, we measured the absorbance of the beads in transmission mode. Then, we placed the beads underneath fresh brain tissue, and using the same procedure described above, we computed $I_{0}$ for our “epi mode” approach using the beads as reference (instead of cell nuclei as described above). We performed this bead validation only at 255 nm, as the beads absorb substantially less at 280 nm [93], which could lead to inaccurate results. A statistical comparison between the $I_{0}$ values calculated from polystyrene beads and the nuclei in mouse brain in individual FOVs is presented in Fig. S3 and revealed no statistically significant differences.

With the calibrated $I_{0}$ at 255 and 280 nm, we then calculated the concentrations of protein and nucleic acid *at* each pixel (in molarity, M) by solving Eq. (1) at each wavelength. Multiplying these concentrations by the magnified pixel area (~0.062 μm^2^) and an effective path length (defined as the average DOF) provided the quantities in moles. From these values, the masses of protein and nucleic acid were determined based on their respective molar masses. These masses are defined as the mass contained within the volume represented by each pixel, projected through the DOF.

### Quantification of the autofluorescence quantum yield of tryptophan

The quantification process for the quantum yield of tryptophan in transmission UV microscopy has been thoroughly characterized in [45]. In this study, we employed a similar approach, with minor modifications to account for the epi-illumination setup. The autofluorescence intensity at each pixel can be expressed as follows:

$$\begin{array}{c} I_{\mathrm{AF}}\left(x,y\right)=\\ \left(\frac{T_{\mathrm{AF}}}{T_{\text{trans}}}\right)\left(\frac{{\mathrm{TE}}_{\mathrm{AF}}}{{\mathrm{TE}}_{\text{trans}}}\right)\left(\frac{{\text{sin}}^{-1}\left(\mathrm{NA}\right)}{2\cdot \pi }\right)\times q\left(x,y\right)\times I_{0}\left(x,y\right)\times \left(1-10^{-A^{\mathrm{Trp}}}\right) \end{array}$$ (4)

and

$$A^{\mathrm{Trp}}=c^{\text{protein}}\times 2\cdot l_{255}\times \varepsilon_{255}^{\mathrm{Trp}}\times N_{\mathrm{aa}}\times F_{\mathrm{Trp}}$$ (5)

where $I_{\mathrm{AF}}\left(x,y\right)$ is the autofluorescence intensity of tryptophan at each pixel (357F images). $T_{\mathrm{AF}}$ and $T_{\text{trans}}$ are the exposure times used in capturing the autofluorescence image and the transmission image, respectively; ${\mathrm{TE}}_{\mathrm{AF}}$ and ${\mathrm{TE}}_{\text{trans}}$ are the transmission efficiency of the bandpass filters for autofluorescence and transmission at 357 and 255 nm, respectively; NA is the numerical aperture of the objective lens; $q\left(x,y\right)$ is the autofluorescence quantum yield of tryptophan at each pixel; $I_{0}\left(x,y\right)$ represents the pixel values of the initial input light intensity at the sample plane within the FOV; $A^{\mathrm{Trp}}$ is the absorbance due to tryptophan; $c^{\text{protein}}$ is the concentration of protein calculated for the mass map; $l_{255}$ is the DOF of the objective at 255 nm (path length); $\varepsilon_{255}^{\mathrm{Trp}}$ is the molar extinction coefficient of tryptophan at 255 nm; $N_{\mathrm{aa}}$ is the average number of amino acids per protein; and $F_{\mathrm{Trp}}$ is the average tryptophan frequency in amino acids. We estimated $I_{0}\left(x,y\right)$ by illuminating a blank quartz slide with a 255-nm LED in transmission mode, at the same power on the sample stage as in the epi-DUV setup (~7.1 mW). This approach approximates the amount of light power entering the tissue for the 255-nm illumination in epi-mode. Note that this $I_{0}\left(x,y\right)$ differs from $I_{0,\lambda }$, with the latter representing the intensity at the diffusive layer, which is used to compute mass maps. This distinction also deviates from the definition in [45] for transmission mode, where the same $I_{0}\left(x,y\right)$ was used to compute both the quantities of mass and quantum yield. The difference arises from the assumption that the initial light intensity is substantially attenuated before reaching the diffusive layer and that only a small portion of the scattered photons from the diffusive layer is collected, whereas nearly all the initial light contributes to potential tryptophan absorption and subsequent autofluorescence. This assumption is further supported by the fact that we used the same exposure time for both the absorption and autofluorescence images in epi-DUV, while in transmission-mode UV absorption microscopy, the exposure time is much shorter than that used for autofluorescence [45].

In the equation for $A^{\mathrm{Trp}}$, a factor of 2 is applied to the path length, reflecting the assumption that the 255-nm UV penetration depth that generates detected autofluorescence is approximately twice the depth for the diffusely back-reflected 255-nm light that is detected in the absorption image (which is comparable to the DOF). This is because the latter undergoes a round trip, meaning it must travel back from the diffusive layer without being absorbed. Consequently, it is reasonable to assume that the light at the diffusive layer can also be scattered into deeper regions in the tissue at approximately the same distance. The exact penetration depth should ideally be modeled using more rigorous approaches such as Monte Carlo (MC) simulations. However, estimating a UV penetration depth at approximately twice the DOF of our objective lens (~12 μm) is a reasonable assumption, given the relatively extreme oblique illumination in our setup and previously reported data on UV penetration depths [25,53,94]. All 255-nm light within this penetration depth can potentially be absorbed by tryptophan, thereby contributing to tryptophan autofluorescence. The mismatch between the UV sectioning thickness and the DOF is further supported by our 357F images, which appear noticeably blurrier compared to the 255A and 280A images.

With the above assumptions and the quantities described in Table 1, $q\left(x,y\right)$ can be computed for each pixel according to Eq. (4).

### Comparison of quantitative values with previously reported data

In Fig. 3, our measured values for nucleic acid mass, protein mass, and quantum yield are up to 30 fg, 450 fg, and 15% per 0.062 μm^2^. This pixel size is 0.62 times the pixel size used in ref. [41] (0.1 μm^2^), where nucleic acid mass and protein mass of a live neutrophil were reported as up to 80 and 1,000 fg, respectively. Adjusting for the pixel size difference, these values correspond to nucleic acid and protein masses of up to 50 and 620 fg, respectively, which align well with our results.

In another study [45], our pixel size is 1.72 times larger than their pixel size (0.036 μm^2^), where nucleic acid mass, protein mass, and the quantum yield of tryptophan for a live mouse macrophage cell were reported as up to 10 fg, 100 fg, and 12%, respectively. Scaling for the pixel size difference, these values correspond to nucleic acid mass, protein mass, and quantum yield of 17 fg, 170 fg, and 20%, respectively, which again align well with the range of our reported values.

Despite differences in cell types, our measured quantitative values show close agreement with those reported in the literature.

### Pseudo-colorization with 255A

The purpose of pseudo-colorization is to generate realistic H&E-like images with high nuclear contrast. The density and morphology of nuclei are among the most critical diagnostic features and are often sufficient for pathologists to make accurate diagnoses. The 255A images were selected for this transformation because they provide the highest nuclear contrast. A straightforward approach to performing the color transformation involves matching the histogram of the 255A image to that of a reference H&E image. This ensures that the dark nuclei in the 255A image correspond to the purplish nuclei in the H&E image, while the surrounding structures (that do not contain high nucleic acid concentrations) adopt a pinkish hue. This process can be efficiently executed using the built-in MATLAB function “imhistmatch”, which adjusts (shifts and stretches) the histogram of the 255A image to align with each of the RGB channels of the reference H&E image. The adjusted 3 channels were then concatenated to produce a pseudo-H&E image. This process is exceptionally simple and fast and does not require post-processing while successfully generating realistic H&E-like images with clear nuclear contrast. It is important to note that for consistent, high-quality histogram matching, the flat-field correction of the 255A image is critical, but the images do not need to be quantitative, i.e., normalizing by *I*_0_ is not necessary.

## Data Availability

Data underlying the results presented in this paper are not publicly available at this time but may be obtained from the authors upon reasonable request.

## Supplementary Materials

Supplementary 1 Texts S1 to S4 Figs. S1 to S7

## References

[1] Bancroft JD, Gamble M. *Theory and practice of histological techniques*. 8th ed. Amsterdam (Netherlands): Elsevier; 2018.

[2] Fleming FJ, Hill ADK, Mc Dermott EW, O’Doherty A, O’Higgins NJ, Quinn CM. Intraoperative margin assessment and re-excision rate in breast conserving surgery. Eur J Surg Oncol. 2004;30(3):233–237. 10.1016/j.ejso.2003.11.008 15028301

[3] Maloney BW, McClatchy DM, Pogue BW, Paulsen KD, Wells WA, Barth RJ. Review of methods for intraoperative margin detection for breast conserving surgery. J Biomed Opt. 2018;23(10):100901. 10.1117/1.JBO.23.10.100901 PMC6210801 30369108

[4] Yong WH, editor. Biobanking: Methods and protocols. In: *Methods in molecular biology*. Vol. 1897. New York: Springer; 2019.

[5] Al-Mulla F, editor. Formalin-fixed paraffin-embedded tissues: Methods and protocols. In: *Methods in molecular biology*. Vol. 724. Totowa (NJ): Humana Press; 2011.

[6] Rastogi V, Puri N, Arora S, Kaur G, Yadav L, Sharma R. Artefacts: A diagnostic dilemma—A review. J Clin Diagn Res. 2013;7(10):2408–2413. 10.7860/JCDR/2013/6170.3541 PMC3843421 24298546

[7] Taxy JB. Frozen section and the surgical pathologist: A point of view. Arch Pathol Lab Med. 2009;133(7):1135–1138. 10.5858/133.7.1135 19642740

[8] Kang J, Song I, Kim H, Kim H, Lee S, Choi Y, Chang HJ, Sohn DK, Yoo H. Rapid tissue histology using multichannel confocal fluorescence microscopy with focus tracking. Quant Imaging Med Surg. 2018;8(9):884–893. 10.21037/qims.2018.09.18 PMC6218212 30505717

[9] Rajadhyaksha M, Menaker G, Flotte T, Dwyer PJ, González S. Confocal examination of nonmelanoma cancers in thick skin excisions to potentially guide Mohs micrographic surgery without frozen histopathology. J Invest Dermatol. 2001;117(5):1137–1143. 10.1046/j.0022-202x.2001.01524.x 11710924

[10] Abeytunge S, Larson B, Peterson G, Morrow M, Rajadhyaksha M, Murray MP. Evaluation of breast tissue with confocal strip-mosaicking microscopy: A test approach emulating pathology-like examination. J Biomed Opt. 2017;22(3): Article 034002. 10.1117/1.JBO.22.3.034002 PMC5361391 28327961

[11] Dobbs JL, Ding H, Benveniste AP, Kuerer HM, Krishnamurthy S, Yang W, Richards-Kortum R. Feasibility of confocal fluorescence microscopy for real-time evaluation of neoplasia in fresh human breast tissue. J Biomed Opt. 2013;18(10): Article 106016. 10.1117/1.JBO.18.10.106016 24165742

[12] Olson E, Levene MJ, Torres R. Multiphoton microscopy with clearing for three dimensional histology of kidney biopsies. Biomed Opt Express. 2016;7(8):3089. 10.1364/BOE.7.003089 PMC4986816 27570700

[13] Tao YK, Shen D, Sheikine Y, Ahsen OO, Wang HH, Schmolze DB, Johnson NB, Brooker JS, Cable AE, Connolly JL, et al. Assessment of breast pathologies using nonlinear microscopy. Proc Natl Acad Sci USA. 2014;111(43):15304–15309. 10.1073/pnas.1416955111 PMC4217415 25313045

[14] Cahill LC, Giacomelli MG, Yoshitake T, Vardeh H, Faulkner-Jones BE, Connolly JL, Sun C-K, Fujimoto JG. Rapid virtual hematoxylin and eosin histology of breast tissue specimens using a compact fluorescence nonlinear microscope. Lab Investig. 2018;98(1):150–160. 10.1038/labinvest.2017.116 PMC5752596 29131161

[15] Jain M, Robinson BD, Wu B, Khani F, Mukherjee S. Exploring multiphoton microscopy as a novel tool to differentiate chromophobe renal cell carcinoma from oncocytoma in fixed tissue sections. Arch Pathol Lab Med. 2018;142(3):383–390. 10.5858/arpa.2017-0056-OA 29219617

[16] Sarri B, Poizat F, Heuke S, Wojak J, Franchi F, Caillol F, Giovannini M, Rigneault H. Stimulated Raman histology: One to one comparison with standard hematoxylin and eosin staining. Biomed Opt Express. 2019;10(10):5378. 10.1364/BOE.10.005378 PMC6788596 31646052

[17] Hollon TC, Pandian B, Adapa AR, Urias E, Save AV, Khalsa SSS, Eichberg DG, D’Amico RS, Farooq ZU, Lewis S, et al. Near real-time intraoperative brain tumor diagnosis using stimulated Raman histology and deep neural networks. Nat Med. 2020;26(1):52–58. 10.1038/s41591-019-0715-9 PMC6960329 31907460

[18] Orringer DA, Pandian B, Niknafs YS, Hollon TC, Boyle J, Lewis S, Garrard M, Hervey-Jumper SL, Garton HJL, Maher CO, et al. Rapid intraoperative histology of unprocessed surgical specimens via fibre-laser-based stimulated Raman scattering microscopy. Nat Biomed Eng. 2017;1(2):0027. 10.1038/s41551-016-0027 PMC5612414 28955599

[19] Saar BG, Freudiger CW, Reichman J, Stanley CM, Holtom GR, Xie XS. Video-rate molecular imaging in vivo with stimulated Raman scattering. Science. 2010;330(6009):1368–1370. 10.1126/science.1197236 PMC3462359 21127249

[20] Alfonso-García A, Mittal R, Lee ES, Potma EO. Biological imaging with coherent Raman scattering microscopy: A tutorial. J Biomed Opt. 2014;19(7): Article 071407. 10.1117/1.JBO.19.7.071407 PMC4019423 24615671

[21] Beard P. Biomedical photoacoustic imaging. Interface Focus. 2011;1(4):602–631. 10.1098/rsfs.2011.0028 PMC3262268 22866233

[22] Wang LV, Hu S. Photoacoustic tomography: In vivo imaging from organelles to organs. Science. 2012;335(6075):1458–1462. 10.1126/science.1216210 PMC3322413 22442475

[23] Yao DK, Chen R, Maslov K, Zhou Q, Wang LV. Optimal ultraviolet wavelength for in vivo photoacoustic imaging of cell nuclei. J Biomed Opt. 2012;17(5): Article 056004. 10.1117/1.JBO.17.5.056004 PMC3602808 22612127

[24] Shi J, Wong TTW, He Y, Li L, Zhang R, Yung CS, Hwang J, Maslov K, Wang LV. High-resolution, high-contrast mid-infrared imaging of fresh biological samples with ultraviolet-localized photoacoustic microscopy. Nat Photonics. 2019;13(9):609–615. 10.1038/s41566-019-0441-3 PMC6705424 31440304

[25] Wong TTW, Zhang R, Hai P, Zhang C, Pleitez MA, Aft RL, Novack DV, Wang LV. Fast label-free multilayered histology-like imaging of human breast cancer by photoacoustic microscopy. Sci Adv. 2017;3(5): Article e1602168. 10.1126/sciadv.1602168 PMC5435415 28560329

[26] Haven NJM, Bell KL, Kedarisetti P, Lewis JD, Zemp RJ. Ultraviolet photoacoustic remote sensing microscopy. Opt Lett. 2019;44(14):3586. 10.1364/OL.44.003586 31305578

[27] Martell MT, Haven NJM, Cikaluk BD, Restall BS, McAlister EA, Mittal R, Adam BA, Giannakopoulos N, Peiris L, Silverman S, et al. Deep learning-enabled realistic virtual histology with ultraviolet photoacoustic remote sensing microscopy. Nat Commun. 2023;14(1):5967. 10.1038/s41467-023-41574-2 PMC10519961 37749108

[28] Cikaluk BD, Restall BS, Haven NJM, Martell MT, McAlister EA, Zemp RJ. Rapid ultraviolet photoacoustic remote sensing microscopy using voice-coil stage scanning. Opt Express. 2023;31(6):10136. 10.1364/OE.481313 37157568

[29] Fereidouni F, Harmany ZT, Tian M, Todd A, Kintner JA, McPherson JD, Borowsky AD, Bishop J, Lechpammer M, Demos SG, et al. Microscopy with ultraviolet surface excitation for rapid slide-free histology. Nat Biomed Eng. 2017;1(12):957–966. 10.1038/s41551-017-0165-y PMC6223324 31015706

[30] Ang JLY, Tan KH, Yong ASK, Tan CWX, Kng JSJ, Tan CJJ, Soh RHK, Tan JYH, Liang K. Multi-scale tissue fluorescence mapping with fiber optic ultraviolet excitation and generative modeling. Optica. 2024;11(5):673–685.

[31] Schlichenmeyer TC, Wang M, Elfer KN, Brown JQ. Video-rate structured illumination microscopy for high-throughput imaging of large tissue areas. Biomed Opt Express. 2014;5(2):366. 10.1364/BOE.5.000366 PMC3920869 24575333

[32] Wang M, Kimbrell HZ, Sholl AB, Tulman DB, Elfer KN, Schlichenmeyer TC, Lee BR, Lacey M, Brown JQ. High-resolution rapid diagnostic imaging of whole prostate biopsies using video-rate fluorescence structured illumination microscopy. Cancer Res. 2015;75(19):4032–4041. 10.1158/0008-5472.CAN-14-3806 PMC4592466 26282168

[33] Kaza N, Ojaghi A, Robles FE. Virtual staining, segmentation, and classification of blood smears for label-free hematology analysis. BME Front. 2022;2022:9853606. 10.34133/2022/9853606 PMC10521747 37850166

[34] Soltani S, Ojaghi A, Robles FE. Deep UV dispersion and absorption spectroscopy of biomolecules. Biomed Opt Express. 2019;10(2):487. 10.1364/BOE.10.000487 PMC6377894 30800494

[35] Soltani S, Ojaghi A, Qiao H, Kaza N, Li X, Dai Q, Osunkoya AO, Robles FE. Prostate cancer histopathology using label-free multispectral deep-UV microscopy quantifies phenotypes of tumor aggressiveness and enables multiple diagnostic virtual stains. Sci Rep. 2022;12(1):9329. 10.1038/s41598-022-13332-9 PMC9167293 35665770

[36] Soltani S, Cheng B, Osunkoya AO, Robles FE. Deep UV microscopy identifies prostatic basal cells: An important biomarker for prostate cancer diagnostics. BME Front. 2022;2022:9847962. 10.34133/2022/9847962 PMC10521648 37850167

[37] Ojaghi A, Kendall Williams E, Kaza N, Gorti V, Choi H, Torey J, Wiley T, Turner B, Jackson S, Park S, et al. Label-free deep-UV microscopy detection and grading of neutropenia using a passive microfluidic device. Opt Lett. 2022;47(22):6005. 10.1364/OL.472691 37219158

[38] Gorti V, Subramanian AR, Ojaghi A, Nsonwu-Farley J, Tran R, Williams EK, Torres O, Aljudi A, Aumann W, Robles FE. Rapid, point-of-care bone marrow aspirate adequacy assessment via deep ultraviolet microscopy. Lab Investig. 2025;105(5): Article 104102. 10.1016/j.labinv.2025.104102 PMC12097932 39909141

[39] Gorti V, McCubbins K, Houston D, Silva Trenkle AD, Holberton A, Serafini CE, Wood L, Kwong G, Robles FE. Quantifying UV-induced photodamage for longitudinal live-cell imaging applications of deep-UV microscopy. Biomed Opt Express. 2025;16(1):208. 10.1364/BOE.544778 PMC11729288 39816147

[40] Gorti V, Kaza N, Williams EK, Lam WA, Robles FE. Compact and low-cost deep-ultraviolet microscope system for label-free molecular imaging and point-of-care hematological analysis. Biomed Opt Express. 2023;14(3):1245. 10.1364/BOE.482294 PMC10026585 36950241

[41] Ojaghi A, Fay ME, Lam WA, Robles FE. Ultraviolet hyperspectral interferometric microscopy. Sci Rep. 2018;8(1):9913. 10.1038/s41598-018-28208-0 PMC6028425 29967322

[42] Si M, Dai W, Wong IHM, Zhang Y, Ma TKF, Ng PW, Li KH, Wong SMS, Ngan JHK, Wong TTW. Autofluorescence imaging and virtual histological staining of human prostate sections for cancer diagnosis. Adv Intell Syst. 2025;7(11):2401081.

[43] Gorti V, Si M, Taylor N, Cicerone M, Robles FE, Deep-ultraviolet microscopy reveals biomolecular spatiotemporal intracellular dynamics. bioRxiv. 2026. 10.64898/2026.08.02.742310

[44] Ojaghi A, Carrazana G, Caruso C, Abbas A, Myers DR, Lam WA, Robles FE. Label-free hematology analysis using deep-ultraviolet microscopy. Proc Natl Acad Sci USA. 2020;117(26):14779–14789. 10.1073/pnas.2001404117 PMC7334528 32561645

[45] Zeskind BJ, Jordan CD, Timp W, Trapani L, Waller G, Horodincu V, Ehrlich DJ, Matsudaira P. Nucleic acid and protein mass mapping by live-cell deep-ultraviolet microscopy. Nat Methods. 2007;4(7):567–569. 10.1038/nmeth1053 17546037

[46] Ye S, Zou J, Huang C, Xiang F, Wen Z, Wang N, Yu J, He Y, Liu P, Mei X, et al. Rapid and label-free histological imaging of unprocessed surgical tissues via dark-field reflectance ultraviolet microscopy. iScience. 2023;26(1): Article 105849. 10.1016/j.isci.2022.105849 PMC9839964 36647380

[47] Kumamoto Y, Taguchi A, Kawata S. Deep-ultraviolet biomolecular imaging and analysis. Adv Opt Mater. 2019;7(5):1801099.

[48] Li C, Pitsillides C, Runnels JM, Côté D, Lin CP. Multiphoton microscopy of live tissues with ultraviolet autofluorescence. IEEE J Sel Top Quantum Electron. 2010;16(3):516–523.

[49] Lakowicz JR, Masters BR. Principles of fluorescence spectroscopy, third edition. J Biomed Opt. 2008;13(2): Article 029901.

[50] Liu H, Zhang H, Jin B. Fluorescence of tryptophan in aqueous solution. Spectrochim Acta A Mol Biomol Spectrosc. 2013;106:54–59. 10.1016/j.saa.2012.12.065 23357679

[51] Cheong WF, Prahl SA, Welch AJ. A review of the optical properties of biological tissues. IEEE J Quantum Electron. 1990;26(12):2166–2185.

[52] Ritz J, Roggan A, Isbert C, Müller G, Buhr HJ, Germer C. Optical properties of native and coagulated porcine liver tissue between 400 and 2400 nm. Lasers Surg Med. 2001;29(3):205–212. 10.1002/lsm.1134 11573221

[53] Tsia K, editor. *Understanding biophotonics: Fundamentals, advances, and applications*. Jenny Stanford Publishing; 2016.

[54] Martins IS, Silva HF, Lazareva EN, Chernomyrdin NV, Zaytsev KI, Oliveira LM, Tuchin VV. Measurement of tissue optical properties in a wide spectral range: A review invited. Biomed Opt Express. 2023;14(1):249. 10.1364/BOE.479320 PMC9841994 36698664

[55] Nogueira MS, Maryam S, Amissah M, Lu H, Lynch N, Killeen S, O’Riordain M, Andersson-Engels S. Evaluation of wavelength ranges and tissue depth probed by diffuse reflectance spectroscopy for colorectal cancer detection. Sci Rep. 2021;11(1):798. 10.1038/s41598-020-79517-2 PMC7804163 33436684

[56] Yusoff MNS, Jaafar MS. Effect of multi-design skin model and characteristic on Monte Carlo simulation of light-skin diffuse reflectance spectra. J Teknol. 2016;78(9):135–142.

[57] Wisotzky EL, Uecker FC, Dommerich S, Hilsmann A, Eisert P, Arens P. Determination of optical properties of human tissues obtained from parotidectomy in the spectral range of 250 to 800 nm. J Biomed Opt. 2019;24(12):1. 10.1117/1.JBO.24.12.125001 PMC6890295 31797647

[58] Gonçalves TM, Martins IS, Silva HF, Tuchin VV, Oliveira LM. Spectral optical properties of rabbit brain cortex between 200 and 1000 nm. Photochem. 2021;1(2):190–208.

[59] Yoshitake T, Giacomelli MG, Quintana LM, Vardeh H, Cahill LC, Faulkner-Jones BE, Connolly JL, Do D, Fujimoto JG. Rapid histopathological imaging of skin and breast cancer surgical specimens using immersion microscopy with ultraviolet surface excitation. Sci Rep. 2018;8(1):4476. 10.1038/s41598-018-22264-2 PMC5852098 29540700

[60] Sidman RL, Angevine JB, Pierce ET. *High resolution mouse brain atlas—Methods*. Cambridge (MA): Harvard Medical School. http://www.hms.harvard.edu/research/brain/methods.html

[61] Tekko T, Lakspere T, Allikalt A, End J, Kõlvart KR, Jagomäe T, Terasmaa A, Philips M-A, Visnapuu T, Väärtnõu F, et al. Wfs1 is expressed in dopaminoceptive regions of the amniote brain and modulates levels of D1-like receptors. PLOS ONE. 2017;12(3): Article e0172825. 10.1371/journal.pone.0172825 PMC5436468 28267787

[62] Suzuki S, Mori A. Regional distribution of tyrosine, tryptophan, and their metabolites in the brain of epileptic El mice. Neurochem Res. 1992;17(7):693–698. 10.1007/BF00968007 1407265

[63] Ching-Roa VD, Huang CZ, Giacomelli MG. Improved microscopy with ultraviolet surface excitation (MUSE) using high-index immersion illumination. Biomed Opt Express. 2021;12(10):6461. 10.1364/BOE.435520 PMC8547983 34745749

[64] Ruifrok AC, Johnston DA. Quantification of histochemical staining by color deconvolution. Anal Quant Cytol Histol. 2001;23:291–299. 11531144

[65] Waehneldt TV, Shooter EM. A comparison of the protein composition of the brains of four rodents. Brain Res. 1973;57(2):361–371. 10.1016/0006-8993(73)90142-x 4722060

[66] Fernandez MK, Sinha M, Renz M. Differential intracellular protein distribution in cancer and normal cells—Beta-catenin and CapG in gynecologic malignancies. Cancer. 2022;14(19):4788. 10.3390/cancers14194788 PMC9561979 36230711

[67] Dallaire F, Lee SS, Guiot M-C, Trifiro MA, Petrecca K, Leblond F. Label-free wide-field imaging of brain tumors using near-infrared endogenous fluorescence and reflectance normalization. J Biomed Opt. 2026;31(8): Article 086005. 10.1117/1.JBO.31.8.086005 PMC13463984 42592302

[68] Burr GO, Miller ES. Ultraviolet absorption spectra of fatty acids and their application to chemical problems. Chem Rev. 1941;29(2):419–438.

[69] Angioni E, Lercker G, Frega NG, Carta G, Melis MP, Murru E, Spada S, Banni S. UV spectral properties of lipids as a tool for their identification. Eur J Lipid Sci Technol. 2002;104(1):59–64.

[70] Reiss R, Faccio G, Thöny-Meyer L, Richter M. Cloning, expression and biochemical characterization of the cholesterol oxidase CgChoA from *Chryseobacterium gleum*. BMC Biotechnol. 2014;14(1):46. 10.1186/1472-6750-14-46 PMC4053396 24885249

[71] Zhao Y, Yang Y, Loscalzo J. Real time measurement of metabolic states in living cells using genetically-encoded NADH sensors. Methods Enzymol. 2014;542:349–367. 10.1016/B978-0-12-416618-9.00018-2 PMC4189816 24862275

[72] Zhang Q, Piston DW, Goodman RH. Regulation of corepressor function by nuclear NADH. Science. 2002;295(5561):1895–1897. 10.1126/science.1069300 11847309

[73] Elston CW, Ellis IO. Pathological prognostic factors in breast cancer. I. The value of histological grade in breast cancer: Experience from a large study with long-term follow-up. Histopathology. 1991;19(5):403–410. 10.1111/j.1365-2559.1991.tb00229.x 1757079

[74] Kumar V, Abbas AK, Aster JC, Turner JR, Perkins JA, Robbins SL, Cotran RS, editors. *Robbins & Cotran pathologic basis of disease*. 10th ed. Philadelphia (PA): Elsevier; 2021.

[75] Li Z, Casteleiro Costa P, Serafini C, Bharadwaj S, Guang Z, Robles FE. Experimental assessment of the optical transfer function for quantitative oblique back illumination microscopy (qOBM). Opt Express. 2025;33(3):5088. 10.1364/OE.547477 PMC12011374 40797810

[76] Zheng T, Adi W, Campagnola PJ, Yesilkoy F. Fix or freeze? Spectral differences arising from tissue preparation in chemical imaging. bioRxiv. 2025. 10.1101/2025.11.19.689284 PMC13177295 42148970

[77] Zhang Y, Kang L, Wong IHM, Dai W, Li X, Chan RCK, Hsin MKY, Wong TTW. High-throughput, label-free and slide-free histological imaging by computational microscopy and unsupervised learning. Adv Sci. 2022;9(2):2102358. 10.1002/advs.202102358 PMC8805566 34747142

[78] Li J, Garfinkel J, Zhang X, Wu D, Zhang Y, De Haan K, Wang H, Liu T, Bai B, Rivenson Y, et al. Biopsy-free in vivo virtual histology of skin using deep learning. Light Sci Appl. 2021;10(1):233. 10.1038/s41377-021-00674-8 PMC8602311 34795202

[79] Wang X, Ye Q, Xie D, Wang Y, Song W. Integrated ultraviolet photoacoustic and spectroscopic autofluorescence microscopy for label-free histological and molecular imaging. Appl Phys Lett. 2025;127(20): Article 203702.

[80] Boktor M, Tweel JED, Ecclestone BR, Ye JA, Fieguth P, Haji Reza P. Multi-channel feature extraction for virtual histological staining of photon absorption remote sensing images. Sci Rep. 2024;14(1):2009. 10.1038/s41598-024-52588-1 PMC10805725 38263394

[81] Ecclestone BR, Simmons JAT, Tweel JED, Kaur C, Hajiahmadi A, Haji P. Photon absorption remote sensing (PARS): A comprehensive approach to label-free absorption microscopy across biological scales. arXiv. 2024. 10.48550/arXiv.2403.04229

[82] Abraham TM, Costa PC, Serafini CE, Guang Z, Zhang Z, Neill S, Olson JJ, Levenson R, Robles FE. Label- and slide-free tissue histology using 3D epi-mode quantitative phase imaging and virtual hematoxylin and eosin staining. Optica. 2023;10(12):1605. 10.1364/optica.502859 PMC11620277 39640229

[83] Ledwig P, Robles FE. Epi-mode tomographic quantitative phase imaging in thick scattering samples. Biomed Opt Express. 2019;10(7):3605. 10.1364/BOE.10.003605 PMC6640824 31360607

[84] Ledwig P, Robles FE. Quantitative 3D refractive index tomography of opaque samples in epi-mode. Optica. 2021;8(1):6. 10.1364/optica.410135 PMC8341081 34368406

[85] Ledwig P, Sghayyer M, Kurtzberg J, Robles FE. Dual-wavelength oblique back-illumination microscopy for the non-invasive imaging and quantification of blood in collection and storage bags. Biomed Opt Express. 2018;9(6):2743. 10.1364/BOE.9.002743 PMC6154191 30258687

[86] Costa PC, Guang Z, Ledwig P, Zhang Z, Neill S, Olson JJ, Robles FE. Towards in-vivo label-free detection of brain tumor margins with epi-illumination tomographic quantitative phase imaging. Biomed Opt Express. 2021;12(3):1621. 10.1364/BOE.416731 PMC7984798 33796377

[87] Casteleiro Costa P, Wang B, Filan C, Bowles-Welch A, Yeago C, Roy K, Robles FE. Functional imaging with dynamic quantitative oblique back-illumination microscopy. J Biomed Opt. 2022;27(6): Article 066502. 10.1117/1.JBO.27.6.066502 PMC9243522 35773755

[88] Zhang Y, Huang B, Dai W, Kang L, Tsang VTC, Wu J, Lo CTK, Wong TTW. Label-free and non-destructive histology of unprocessed biological tissues with ultraviolet single-plane illumination microscopy. APL Photonics. 2024;9(1): Article 016116.

[89] Wu J, Dai W, Lo CTK, Tsui LWK, Wong TTW. High-throughput, nondestructive, and low-cost histological imaging with deep-learning-assisted UV microscopy. Adv Imaging. 2024;1(2): Article 021001.

[90] Jin L, Tang Y, Coole JB, Tan MT, Zhao X, Badaoui H, Robinson JT, Williams MD, Vigneswaran N, Gillenwater AM, et al. DeepDOF-SE: Affordable deep-learning microscopy platform for slide-free histology. Nat Commun. 2024;15(1):2935. 10.1038/s41467-024-47065-2 PMC10997797 38580633

[91] Ford TN, Chu KK, Mertz J. Phase-gradient microscopy in thick tissue with oblique back-illumination. Nat Methods. 2012;9(12):1195–1197. 10.1038/nmeth.2219 PMC8407656 23103879

[92] Li Z, Casteleiro Costa P, Guang Z, Serafini CE, Robles FE. GAN-based quantitative oblique back-illumination microscopy enables computationally efficient epi-mode refractive index tomography. Biomed Opt Express. 2024;15(8):4764. 10.1364/BOE.528968 PMC11427205 39346989

[93] Li T, Zhou C, Jiang M. UV absorption spectra of polystyrene. Polym Bull. 1991;25(2):211–216.

[94] Meinhardt M, Krebs R, Anders A, Heinrich U, Tronnier H. Wavelength-dependent penetration depths of ultraviolet radiation in human skin. J Biomed Opt. 2008;13(4): Article 044030. 10.1117/1.2957970 19021357

