# Supplementary material for: Label- and Slide-Free Multispectral Quantitative Epi-Illumination Deep-Ultraviolet Microscopy
Source: BME Front. 2026 Oct 30;7:0337. doi: 10.34133/bmef.0337 (PMC13720143; doi:10.34133/bmef.0337)
Supplement: Supplementary file 1 [file bmef.0337.f1.docx]

label- and slide-free multispectral quantitative epi-illumination deep-uv microscopy: supplemental document

**Supporting Information Text S1**

***Note on modelling absorption, scattering in the deep UV region, and epi-DUV effective sectioning:***

We use Beer's law to quantify the absorption images under the assumption that the “diffusive” layer (i.e., the virtual light source within the tissue) is homogeneous and immediately above the surface layer that is analyzed. We argue that these assumptions are reasonable, given that the acquired DUV absorption images do not suffer from significant out-of-focus contributions. We sought to further characterize UV penetration using Monte Carlo (MC) simulations; however, as highlighted in the Results section, the availability of reliable data on DUV scattering and absorption properties of intact tissues is limited and contains significant discrepancies across studies [1–3]. One indicator of this unreliability is that reported values for absorption coefficients ($\sim11-13 {cm}^{-1}$) suggest that, in general, only one absorption event occurs every $\sim770-910$ *µm* in the deep UV range [3]. This estimate is two orders of magnitude higher than the depth of focus of the objective used in this work ($\sim6 \mu m$). If these values were accurate, our images would contain significant out-of-focus background even when using oblique or darkfield illumination (more out-of-focus contributions are observed when using *less* oblique illumination). Similar effects have also been observed using MUSE [4, 5]. This observation suggests that the absorption coefficient in the deep UV range is likely much higher than reported in the literature. While the exact optical properties of tissue in the deep UV range and the precise sectioning thickness remain uncertain, our absorption model is specifically focused on the region between the diffusive layer and the tissue surface. This layer is assumed to be very close to the surface under oblique illumination, with its distance comparable to the depth of focus (DOF) of the objective lens. Combined with the strong absorption behavior of deep UV light, this proximity allows us to approximate light behavior in this regime as predominantly absorptive and effectively modeled by Beer's law.

Here, we provide an estimation of the absorption coefficient ($\mu_{a}$) of fresh mouse brain tissue at 255 nm. First, we calculate the theoretical $\mu_{a}$ of cell nuclei using Eq. 6:

$\mu_{a}= \sum_{i} N_{i}\sigma_{i}$	(S1)

where $N_{i}$ is the density of the *i-th* biomolecules in ${cm}^{-3}$, which can be converted from known average concentrations of nucleic acid and protein in nuclei ($\sim30 mg/ml$, $\sim300 mg/ml$, respectively [6]); $\sigma_{i}$ is the absorption cross-section of the *i-th* biomolecules in ${cm}^{2}$, which can be calculated from the molar extinction coefficient values of nucleic acid and protein at 255 nm. $\mu_{a}$ for nucleic acid is estimated to be $\sim1466{cm}^{-1}$ and that for protein is $\sim472 {cm}^{-1}$. The sum of these values ($\sim1938{cm}^{-1}$) is the total absorption coefficient in cell nuclei and should match the $\mu_{a}$ calculated from Beer’s law in Eq. S2 using the average segmented nuclei intensity and the calibrated $I_{0}$. Note that $I_{0}$ was estimated using (1 literature values of the concentration of nucleic acid and protein in nuclei (which should agree with the theoretical estimate of $\mu_{a}$above, and (2 using polystyrene beads which makes no assumptions about the tissue. Both methods yielded the same value $I_{0}$ (Fig. S3).

$\frac{I}{I_{0}}=e^{-\mu_{a}\cdot z}$	(S2)

Where $I$ is the *average* pixel intensity of segmented cell nuclei at 255 nm (over 50,000 nuclei were used here); $I_{0}$ is the calibrated intensity at the “diffusive” layer (the virtual light source) at 255 nm; $z$ is the path length (DOF of the objective lens) at 255 nm. $\mu_{a}$ is estimated to be $\sim1941{cm}^{-1}$ using these values, which agrees with the total $\mu_{a}$ of the nuclei computed from the absorption of nucleic acid and protein using Eq. S1.

We then substitute $I$ in Eq. 7 with the average pixel intensity of a whole-brain 255A image of a fresh mouse brain, resulting in an estimation of $\mu_{a}=\sim1225 {cm}^{-1}$ for the mouse brain at 255 nm, which is two orders of magnitude higher than the value reported in [3]. Using this value, the absorption length is estimated to be $\sim8.16 \mu m$. Considering the geometry of the tilted illumination source, the absorption length parallel to the optical axis is $\sim6.23 \mu m$, which agrees well with the DOF at 255 nm ($\sim5.84 \mu m$).

**Supporting Information Text S2**

***Note on absorption from other potential biomolecules:***

Native, unoxidized fatty acids (e.g., oleic, stearic, and palmitic acids), which are highly abundant in biological tissues, have a molar extinction coefficient of effectively zero above 250 nm [7] and thus exhibit no measurable absorbance. Generally, tissue lipids only begin to exhibit absorption above 250 nm when they become conjugated under oxidative stress [7, 8]. Cholesterol is another abundant lipid biomolecule; however, because it lacks conjugation, it also has minimal absorbance near 250 nm unless chemically altered [9]. Furthermore, while coenzymes like NADH do have an absorption peak near 260 nm (with a high molar extinction coefficient), their intracellular concentrations are typically in the nanomolar to low micromolar (nM–µM) range [10, 11]. In contrast, the local concentrations of nucleic acids and proteins in cells are in the millimolar (mM) range (tens to hundreds of mg/mL). Therefore, the observed absorption signal is overwhelmingly dominated by nucleic acids and proteins.

Although our mass models at 255 and 280 nm are theoretically robust, residual absorbance from lipids may still occur due to practical hardware limitations, such as the finite optical density (O.D.) and bandwidth of the filters. To further improve the accuracy of these models and introduce greater molecular specificity, future work could incorporate additional wavelengths, specifically those corresponding to the absorption peaks of native lipids [7, 8]. By experimentally determining the molar extinction coefficients of lipids across multiple wavelengths, tissue lipid concentrations could also be quantitatively assessed. More broadly, integrating a wider spectrum of wavelengths would yield a more comprehensive and faithful representation of the molecular composition within intact tissues.

**Supporting Information Text S3**

***Technical comparison between transmission-DUV and epi-DUV:***

While the optical hardware for epi-DUV and transmission-DUV (trans-DUV) is similar, epi-DUV provides the critical advantage of imaging fresh, intact, thick tissue, whereas trans-DUV is strictly limited to thinly sectioned slices. Although quantitative modeling for trans-DUV has been previously established [6], the ability to achieve transmission-like, quantitative image quality directly on thick tissue using our proposed model represents the major technical advance of epi-DUV.

The physical and operational differences between the two modalities are outlined below:

**Signal and Illumination:** Due to the highly attenuating properties of thick tissue in the UV spectrum, epi-DUV requires significantly more illumination power. At equal power, epi-DUV requires approximately 33 times the exposure time (330 ms) compared to trans-DUV (10 ms) to achieve a comparable signal-to-noise ratio (SNR). Interestingly, we note that by using this same exposure time (330 ms) for tryptophan autofluorescence and MUSE (see Fig. S6) as we did for epi-DUV, we were able to achieve a similar SNR across these techniques.

**Background Levels:** Standard background correction in trans-DUV relies on tissue-free regions (e.g., glass/quartz). Because thick tissues lack these "empty" regions, standard correction is impossible for epi-DUV. Instead, our quantitative model assumes the background is a diffusive layer within the tissue exhibiting Lambertian scattering.

**Axial Resolution:** In trans-DUV, the physical thinness of the slide limits out-of-focus contributions. For epi-DUV on thick tissue, the illumination angle must be carefully optimized. As detailed in the main text, a 70° DUV illumination angle effectively removes out-of-focus content, providing a virtual axial sectioning (~6 µm) that matches the microscope’s depth of field. This effective sectioning outperforms that of autofluorescence and MUSE. In autofluorescence, we see significant out of focus contributions (e.g., Fig. 1), while in MUSE we see significantly more crowded nuclei than in epi-DUV (Fig. S6).

Figure S7 provides a side-by-side comparison of a quantitative epi-DUV image (thick, fresh sample) and a quantitative trans-DUV image (thin section) obtained from the same region of a mouse brain. A line profile plotted across a single nucleus in each field of view shows slightly higher contrast in the trans-DUV image. This is likely because the formalin-fixed paraffin-embedded (FFPE) process alters tissue properties and increases scattering, which enhances nuclear contrast—a phenomenon similarly observed in autofluorescence imaging [12]. Despite this, the overall contrast and quantitative attenuation values of the nuclei in both images remain highly comparable. This demonstrates that epi-DUV successfully matches the standard of trans-DUV imaging while operating on arbitrarily thick, unprocessed fresh tissue.

**Supporting Information Text S4**

***Experimental protocol for MUSE imaging:***

Hoechst 33342 (Life Technologies, Eugene, OR, USA) was utilized as the nuclear stain. Following standard MUSE protocols [4], the dye was diluted to 500 µg/mL in phosphate-buffered saline (PBS). Fresh mouse brain and kidney tissue samples, obtained in accordance with the Institutional Animal Care and Use Committee (IACUC) at the Georgia Institute of Technology, were submerged in the Hoechst solution for 10 seconds and subsequently washed briefly with PBS.

MUSE imaging was performed using the same optical setup and tissue mounting procedure as the epi-DUV system, utilizing 280-nm excitation. To capture the Hoechst fluorescence emission, a 425-nm long-pass emission filter was introduced into the detection path. For a more direct comparison between single-channel nuclear stain and epi-DUV, images were acquired using the same monochrome camera and exposure time (330 ms) as employed for the epi-DUV imaging. Results are presented in Fig. S6, and additional discussion comparing MUSE and epi-DUV is included in the main text, Results section “Label-free epi-DUV absorption and autofluorescence imaging of fresh mouse brain.”

Figure S1. Experimental resolution testing using high-resolution USAF test target (Newport). (A) Resolution at 255 nm. (B) Resolution at 280 nm. Line plots corresponding to a resolution of 775 nm (red), 691 nm (green), 615 nm (blue), and 548 nm (purple). These measurements were performed in transmission mode.

**Figure S2. Illumination angle-dependent contrast in a 280A image.** (**A**) Illumination with a light pipe at an angle of ~60° to the optical axis. (**B**) Illumination with a light pipe at an angle of ~45° to the optical axis. (**C**) Illumination with a light pipe at an angle of ~30° to the optical axis.

**Figure S3.** $\boldsymbol{I}_{\boldsymbol{0}}$ **validation for 255A by comparing distributions of** $\boldsymbol{I}_{\boldsymbol{0}}$ **computed from the mouse brain and polystyrene beads.** Welch’s t-test and Wilcoxon rank sum tests were conducted with n = 375 for the brain group and n = 15 for the bead group. No statistically significant differences were observed between the two groups (the significance is defined as $p\leq0.05$).

**Figure S4. Comparison of quantitative values of a kidney region calculated using the mouse brain** $\boldsymbol{I}_{\boldsymbol{0}}$ **and kidney** $\boldsymbol{I}_{\boldsymbol{0}}$**.** (**A**) Row of quantitative values of a region calculated using the mouse brain $I_{0}$. (**B**) Row of quantitative values of the same region calculated using the mouse kidney $I_{0}$. High agreement was observed in protein mass and quantum yield maps, while slight differences were present in the nucleic acid mass map.

**Figure S5. Comparison of quantitative values between the mouse brain and mouse kidney.** (**A**) Row of a region in the mouse brain thalamus. (**B**) Row of a region in the mouse kidney, showing a glomerulus. (**C**) An adjacent H&E slice image of the brain region. (**D**) An adjacent H&E slice image of the kidney region. (**E–G**) Histogram distribution of the two regions in nucleic acid mass, protein mass, and quantum yield, respectively.

**Figure S6. Comparison of epi-DUV (255A) images and MUSE images.** (**A–B**) 255A images of regions in fresh mouse brain. (**C**) 255A image of a region in fresh mouse kidney. (**D–E**) MUSE images of the corresponding regions in mouse brain from images A–B. (**F**) MUSE image of the corresponding region in mouse kidney from image C. (**G–I**) Zoomed-in regions of 255A images A–C, respectively. (**J–L**) Corresponding zoomed-in regions of MUSE images D–F. The results show very similar structure for both modalities, except that nuclei have positive contrast in MUSE instead of negative contrast in epi-DUV, and importantly, the nuclei are overcrowded in MUSE compared to epi-DUV. The nuclear overcrowding in MUSE suggests epi-DUV achieves a thinner optical cross-section, meaning improved axial resolution.

**Figure S7. Comparison of epi-DUV (thick tissue) images and transmission (trans)-DUV (thin slice) images at 255 nm.** (**A**) Trans-DUV image of a region in a thin mouse brain slice. (**B**) Epi-DUV image of the same region in a fresh mouse brain. (**C–D**) Zoomed-in regions of A and B, respectively, revealing a nucleus. (**E**) Line profile along the yellow line plotted in C and D.

**References**

[1]	Martins IS, Silva HF, Lazareva EN, Chernomyrdin NV, Zaytsev KI, Oliveira LM, Tuchin VV, “Measurement of Tissue Optical Properties in a Wide Spectral Range: A Review [Invited].” *Biomedical Optics Express* 14, no. 1 (2023): 249.

[2]	Wisotzky EL, Uecker FC, Dommerich S, Hilsmann A, Eisert P, Arens P, “Determination of Optical Properties of Human Tissues Obtained from Parotidectomy in the Spectral Range of 250 to 800 Nm.” *Journal of Biomedical Optics* 24, no. 12 (2019): 1.

[3]	Gonçalves TM, Martins IS, Silva HF, Tuchin VV, Oliveira LM, “Spectral Optical Properties of Rabbit Brain Cortex between 200 and 1000 Nm.” *Photochem* 1, no. 2 (2021): 190–208.

[4]	Fereidouni F, Harmany ZT, Tian M, Todd A, Kintner JA, McPherson JD, Borowsky AD, Bishop J, Lechpammer M, Demos SG, et al., “Microscopy with Ultraviolet Surface Excitation for Rapid Slide-Free Histology.” *Nature Biomedical Engineering* 1, no. 12 (2017): 957–66.

[5]	Yoshitake T, Giacomelli MG, Quintana LM, Vardeh H, Cahill LC, Faulkner-Jones BE, Connolly JL, Do D, Fujimoto JG, “Rapid Histopathological Imaging of Skin and Breast Cancer Surgical Specimens Using Immersion Microscopy with Ultraviolet Surface Excitation.” *Scientific Reports* 8, no. 1 (2018): 4476.

[6]	Zeskind BJ, Jordan CD, Timp W, Trapani L, Waller G, Horodincu V, Ehrlich DJ, Matsudaira P, “Nucleic Acid and Protein Mass Mapping Supp.” *Nature Methods* 4, no. 7 (2007): 567–69.

[7]	Burr GO, Miller ES, “Ultraviolet Absorption Spectra of Fatty Acids and Their Application to Chemical Problems.” *Chemical Reviews* 29, no. 2 (1941): 419–38.

[8]	Angioni E, Lercker G, Frega NG, Carta G, Melis MP, Murru E, Spada S, Banni S, “UV Spectral Properties of Lipids as a Tool for Their Identification.” *European Journal of Lipid Science and Technology* 104, no. 1 (2002): 59–64.

[9]	Reiss R, Faccio G, Thöny-Meyer L, Richter M, “Cloning, Expression and Biochemical Characterization of the Cholesterol Oxidase CgChoA from Chryseobacterium Gleum.” *BMC Biotechnology* 14, no. 1 (2014): 46.

[10]	Zhao Y, Yang Y, Loscalzo J, “Real Time Measurement of Metabolic States in Living Cells Using Genetically-Encoded NADH Sensors.” *Methods in Enzymology* 542 (2014): 349–67.

[11]	Zhang Q, Piston DW, Goodman RH, “Regulation of Corepressor Function by Nuclear NADH.” *Science* 295, no. 5561 (2002): 1895–97.

[12]	Zhang Y, Kang L, Wong IHM, Dai W, Li X, Chan RCK, Hsin MKY, Wong TTW, “High‐Throughput, Label‐Free and Slide‐Free Histological Imaging by Computational Microscopy and Unsupervised Learning.” *Advanced Science* 9, no. 2 (2022): 2102358.
